# The inflammasome adaptor *pycard* is essential for immunity against *Mycobacterium marinum* infection in adult zebrafish

**DOI:** 10.1242/dmm.052061

**Published:** 2025-03-24

**Authors:** Meri Uusi-Mäkelä, Sanna-Kaisa Emilia Harjula, Maiju Junno, Alina Sillanpää, Reetta Nätkin, Mirja Tellervo Niskanen, Anni Karoliina Saralahti, Matti Nykter, Mika Rämet

**Affiliations:** ^1^ Faculty of Medicine and Health Technology, Tampere University, FI-33014 Tampere, Finland; ^2^ Prostate Cancer Research Center, Faculty of Medicine and Health Technology, Tampere University, FI-33014 Tampere, Finland; ^3^ Tays Cancer Center, Tampere University Hospital, FI-33521 Tampere, Finland

**Keywords:** Zebrafish, Tuberculosis, *Mycobacterium marinum*, Pycard, Inflammasome

## Abstract

Inflammasomes regulate the host response to intracellular pathogens including mycobacteria. We have previously shown that the course of *Mycobacterium marinum* infection in adult zebrafish (*Danio rerio*) mimics the course of tuberculosis in human. To investigate the role of the inflammasome adaptor *pycard* in zebrafish *M. marinum* infection, we produced two zebrafish knockout mutant lines for the *pycard* gene with CRISPR/Cas9 mutagenesis. Although the zebrafish larvae lacking *pycard* developed normally and had unaltered resistance against *M. marinum*, the loss of *pycard* led to impaired survival and increased bacterial burden in the adult zebrafish. Based on histology, immune cell aggregates, granulomas, were larger in *pycard*-deficient fish than in wild-type controls. Transcriptome analysis with RNA sequencing of a zebrafish haematopoietic tissue, kidney, suggested a role for *pycard* in neutrophil-mediated defence, haematopoiesis and myelopoiesis during infection. Transcriptome analysis of fluorescently labelled, *pycard*-deficient kidney neutrophils identified genes that are associated with compromised resistance, supporting the importance of *pycard* for neutrophil-mediated immunity against *M. marinum*. Our results indicate that *pycard* is essential for resistance against mycobacteria in adult zebrafish.

## Teaser

**Summary:** *Mycobacterium marinum* infection in zebrafish mimics tuberculosis. In our model, zebrafish devoid of the inflammasome adaptor gene *pycard* show impaired survival and higher bacterial burden. The expression profile suggests altered neutrophil-mediated defence.

## INTRODUCTION

Every year, ∼10.8[Supplementary-material sup1]million people are diagnosed with tuberculosis, and the disease causes 1.2[Supplementary-material sup1]million deaths (Global Tuberculosis Report, 2024). Tuberculosis is caused by *Mycobacterium tuberculosis* (Mtb)*.* Approximately 410,000 new tuberculosis cases are caused by either rifampicin-resistant or multidrug-resistant Mtb each year (Global Tuberculosis Report, 2024). Owing to the limited efficiency of the Bacillus Calmette–Guérin (BCG) vaccine, new treatments and preventive methods are needed to eliminate tuberculosis (Andersen and Doherty, 2005). Most of the people infected with tuberculosis develop a latent infection, with dormant bacteria (Furin et al., 2019). In a latent infection, the mycobacteria can reside in granulomas without causing symptoms to the host, even for decades. It has been estimated that every fourth person is a carrier of the latent form of tuberculosis (Global Tuberculosis Report, 2024; Houben and Dodd, 2016). The latent infection can reactivate to cause an active disease if the immune system is compromised (Ai et al., 2016).

Tuberculosis is transmitted by the intake of aerosolized bacteria from an infected person (Churchyard et al., 2017). Mtb travel to the lungs and are taken up by alveolar macrophages, where they survive by actively inhibiting the fusion of the phagosome with the lysosome (Goren et al., 1976). Preventing phagosome maturation is just one example of how Mtb manipulate the host's protective pathways (Goldberg et al., 2014; Korb et al., 2016). Mtb are able to inhibit the activation of the inflammasome pathway, which is a key regulator of the post-translational activation of IL-1β and IL-18 (Master et al., 2008; Rastogi et al., 2021). The inflammasome complex activates caspases, which cleave pro-inflammatory cytokines pro-IL-1β and pro-IL-18 into their active form to promote the activation of host immune response (Martinon et al., 2002). The inflammasome complex contains a receptor protein [either NOD-like receptor (NLR) or absent in melanoma 2 (AIM2)] and an adaptor protein [PYC and CARD domain containing (PYCARD; also known as apoptosis-associated speck-like protein containing a CARD (ASC)], which interacts with the interleukin-activating caspases (Martinon et al., 2002). Besides interleukin activation, inflammasomes can also activate gasdermin D, which forms pores in the plasma membrane and can trigger a specialized form of cell death that results in the release of immune activators into the extracellular space, referred to as pyroptosis (Bergsbaken et al., 2009). Thus, inflammasomes serve as a frontline defence mechanism, and several components of the pathway have been shown to be essential for survival in mycobacterial infection (Mayer-Barber et al., 2010; McElvania Tekippe et al., 2010).

The type VII secretion system or early secretory antigenic target secretion of Mtb consists of five (Esx1-5) different protein-secreting pathways essential to mycobacterial pathogenesis (Gröschel et al., 2016). The Esx-1 pathway secretes early secretory antigenic 6 kDa (Esat-6; also known as Esxa) protein, which causes lysis of the phagosomal membranes (Roy et al., 2020). Mtb has been shown to activate the NLRP3/PYCARD inflammasome through the Esat-6 protein, which is essential for mycobacterium-mediated phagosome maturation and mycobacterial virulence (Mishra et al., 2010). Mtb causes damage to the plasma membrane, which induces the potassium efflux-driven activation of the NLRP3 inflammasome in monocytes and macrophages (Beckwith et al., 2020). Intracellular potassium levels suppressing NLRP3 inflammasome responses are regulated by Mtb-induced oxidative stress responsive kinase 1 (OSXR1) expression (Hortle et al., 2022). This can also result in gasdermin D pore formation, pyroptotic cell death and release of IL-1β into the extracellular space (Beckwith et al., 2020). Notably, the attenuated BCG *Mycobacterium bovis* fails to activate the NLRP3 inflammasome, likely due to the lack of the components of the region of difference 1 (RD-1) locus (Dorhoi et al., 2012).

The inflammasome adaptor protein PYCARD connects the receptor protein and the caspase and thus is essential for inflammasome function (Latz et al., 2013). In addition, recent evidence has shown that Pycard has inflammasome-independent roles in immunity, including chemokine regulation and T-helper (Th) cell polarization (Javanmard Khameneh et al., 2019; Taxman et al., 2011). A number of cytokines are regulated by *PYCARD* via inflammasome-dependent and -independent mechanisms during *Porphyromonas gingivalis* infection in THP1 and U937 cell lines (Taxman et al., 2006, 2011). Both the caspase and the Nlrp3 are dispensable for host survival in mouse models of tuberculosis, whereas Pycard knockout mice had impaired survival in an Mtb infection (McElvania Tekippe et al., 2010). The mechanism behind this decreased susceptibility remains unexplored. In some experiments, Pycard has been found to regulate the migration of adaptive immune cells in a Dock2-mediated manner (Ippagunta et al., 2011). Uchiyama et al. (2017) found that the induction of the Th17/Th1 cell response to *Listeria monocytogenes* infection was impaired in *Pycard^−/−^* mice.

The development of novel therapies relies on relevant model systems. The zebrafish (*Danio rerio*) offers a versatile platform to model the human immune system and disease genetics (Lohi et al., 2013). The zebrafish shares over 70% genetic homology with human (Howe et al., 2013). Zebrafish also serve as a valuable model for tuberculosis research, as their natural pathogen, *Mycobacterium marinum*, is closely related to Mtb. The zebrafish/*M. marinum* model is well established (Cronan and Tobin, 2014; Meijer, 2016; Myllymaki et al., 2016, 2018; Saralahti et al., 2020; Tobin and Ramakrishnan, 2008; Tobin et al., 2012). Similar to humans, adult zebrafish infected with *M. marinum* develop an infection with chronic and latent phases with highly structured granulomas (Parikka et al., 2012; Swaim et al., 2007). Using this model, the importance of selected immune genes for resistance against mycobacterial infection can be studied, as shown by us and others (Jia et al., 2024; Harjula et al., 2018; Meijer, 2016; Ojanen et al., 2015, 2019; Parikka et al., 2012). The adult zebrafish has both the innate and adaptive arms of immunity, whereas larval zebrafish rely only on innate immune responses (Lam et al., 2004; Page et al., 2013). Therefore, innate immunity can be studied using zebrafish larvae, and, in turn, the interplay of the innate and adaptive immunities can be studied in adult zebrafish. Zebrafish are also suitable for modelling human inflammasome activation (Frame et al., 2020; Kuri et al., 2017; Li et al., 2019; Tyrkalska et al., 2016, 2017, 2019).

Increasing interest has been directed towards the role of the inflammasome in adaptive immunity, as well as towards the inflammasome-independent role of the adaptor protein Pycard. We have previously shown that *pycard* is upregulated in *M. marinum*-infected adult zebrafish mutants with impaired immunity (Harjula et al., 2020). To further study the role of the inflammasome in the defence against a mycobacterial infection, we generated two knockout *pycard* fish lines and studied their ability to defend themselves against *M. marinum* infection. We found that adult zebrafish devoid of *pycard* are more susceptible to mycobacterial infection than their wild-type (WT) siblings. In addition, RNA sequencing (RNA-seq) of infected adult zebrafish revealed novel genes and pathways relevant for *pycard*-mediated immune defence against a mycobacterial infection, including a number of transcription factors associated with haematopoiesis and myelopoiesis. Further RNA-seq of infected neutrophils revealed differentially expressed genes related to neutrophil function.

## RESULTS

### Mutations generated with CRISPR-Cas9 lead to the elimination of the *pycard* transcript in zebrafish larvae

To study the role of inflammasome signalling in zebrafish, we generated two *pycard* knockout lines with CRISPR-Cas9 technology (Fig. S1A). The mosaic founder fish bearing mutations (AB background) were crossed with WT (TL background) fish, resulting in offspring that were heterozygous for mutations. The F1 heterozygotes carrying a mutation at the end of the first exon of the gene were selected to establish two stable knockout lines, named *pycard^tpu4^* and *pycard^tpu5^* (Fig. S1B-D). Both of the mutations result in a frameshift and a subsequent premature stop codon and termination of transcription (Fig. S1C,D). In both cases, a predicted stop codon results in the deletion of at least the whole caspase recruitment domain (CARD) (Fig. S1). The expression of the *pycard* transcript was measured with quantitative PCR (qPCR) early in development in homozygous knockout and WT siblings. Both of the mutant lines had diminished *pycard* expression (Fig. S1E,F). We considered these lines suitable for studying the loss-of-function phenotype of *pycard* in zebrafish.

### Loss of *pycard* does not impair immunity against *M. marinum* in larval zebrafish

**Fig. 1.**
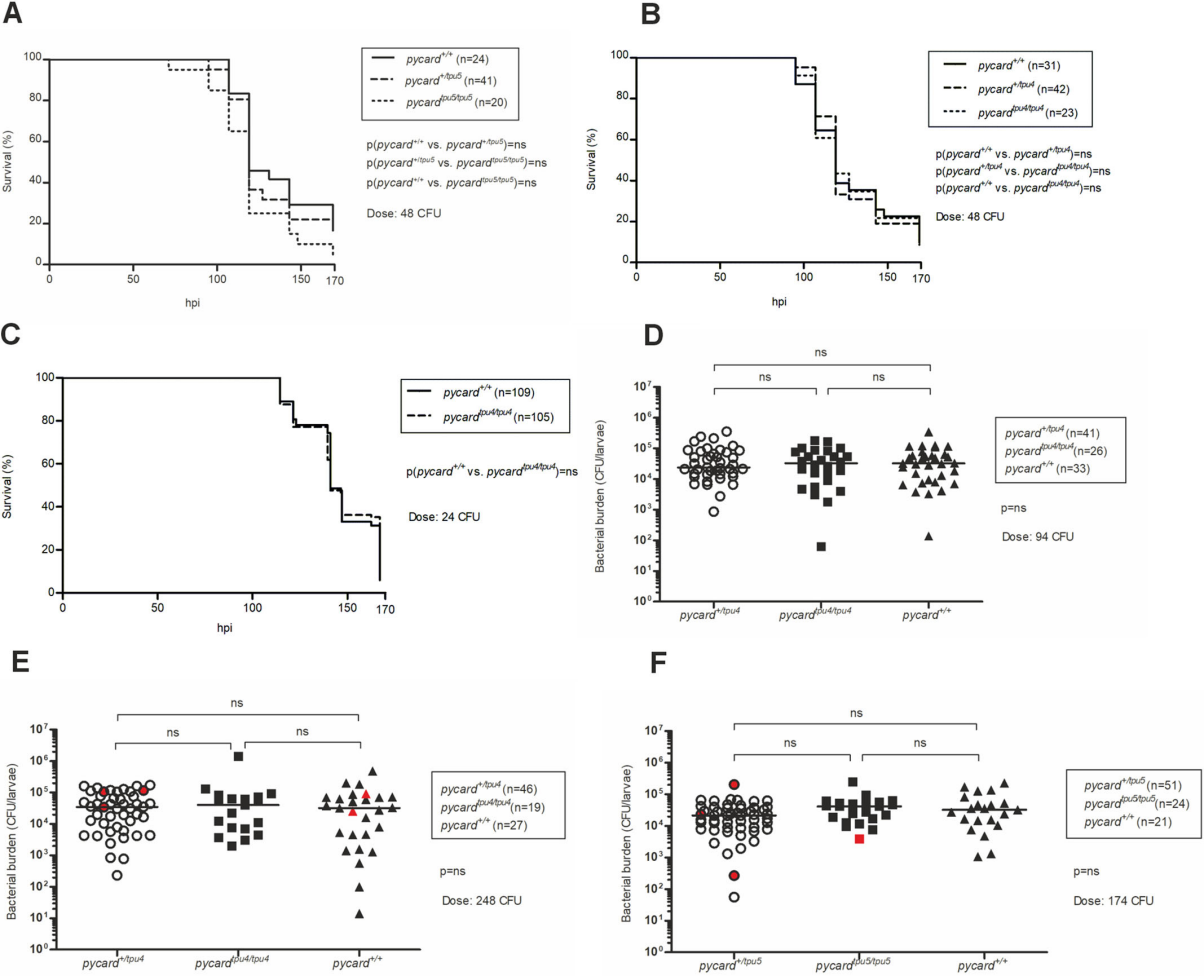
***pycard* deficiency does not affect the survival or bacterial burden in mycobacterial infection in zebrafish larvae.** Embryos were infected with a low dose of *M. marinum* at the two- to eight-cell stage, and their survival was followed for 7 days. (A) F2 generation results for the mutant line *pycard^tpu5^* [average dose, 48 colony-forming units (CFU); range, 17-79 CFU]. End-point survival proportions: *pycard^+/+^*, 16.7%; *pycard^+/tpu5^*, 17.1%; *pycard^tpu5/tpu5^*, 5.0%. hpi, hours post infection. (B) F2 generation results for the mutant line *pycard^tpu4^* (average dose, 48 CFU, range 17-79 CFU). End-point survival proportions: *pycard^+/+^*, 9.7%; *pycard^+/tpu4^*, 11.9%; *pycard^tpu4/tpu4^*, 8.7%). (C) Pooled results of three repeats for the experiment with the F3 generation of *pycard^tpu4^* (average dose, 24 CFU; range, 2-52 CFU). End-point survival proportions: *pycard^+/+^*, 5.8%; *pycard^tpu4/tpu4^*, 12.4%. The average dose was measured from an infection solution plated on 7H10 agar plates. The survival data are presented as a Kaplan–Meier survival curve. Statistical analysis was performed with the log-rank test. (D) Embryos originating from *pycard^+/tpu4^* F4 parents were injected with a low dose of *M. marinum* (average dose, 94 CFU; range, 56-136 CFU) at the two- to 1000-cell stage. Infected larvae were euthanized at 4 days post infection (dpi), and DNA was extracted from the whole larvae. Larvae were genotyped, and bacterial copy number was quantified using quantitative PCR (qPCR) and *M. marinum*-specific primers. (E,F) Larvae originating from *pycard^+/tpu4^* (E) and *pycard^+/tpu5^* (F) F6 parents were infected with *M. marinum* (for *pycard^+/tpu4^*, average dose, 248 CFU; range, 213-283 CFU; for *pycard^+/tpu5^*, average dose, 174 CFU; range, 136-213 CFU) at 2 dpf into blood circulation valley. At 5 dpi, larvae were euthanized, and bacterial copy number was quantified as described in D. Red symbols indicate larvae, which were euthanized according to humane end-point criteria (3 dpi, *n*=1 *pycard^+/tpu5^* and *pycard^+/+^*; 4 dpi, *n*=1 *pycard^+/tpu4^*, *pycard^+/tpu5^* and *pycard^+/+^*; 5 dpi, *n*=1 *pycard^+/tpu4^*) or were dead before the end point (2 dpi, *n*=1 *pycard^+/tpu5^*; 3 dpi, *n*=1 *pycard^tpu5/tpu5^*; 5 dpi, *n*=1 *pycard^+/tpu4^*). Statistical significance was analysed using Kruskal–Wallis test. The line represents the median. ns, not significant.

Zebrafish larvae are protected solely by the innate immune system, as the T and B cells of the adaptive immunity develop only after ∼20 days post fertilization (dpf) (Lam et al., 2004; Page et al., 2013). To investigate whether *pycard* expression is required for resistance against mycobacterial infection in zebrafish larvae, we carried out yolk sack infections at two- to eight-cell stage for both *pycard^tpu4^* and *pycard^tpu5^* (Fig. 1A-C). A low dose of *M. marinum* infection did not result in compromised survival in the *pycard* mutant larvae (Fig. 1A-C). As Pycard could be present in the oocytes from maternal transcripts, the infection experiment was repeated with larvae from homozygous (*pycard^tpu4/tpu4^*) and WT (*pycard^+/+^*) parents. In this setting, *pycard* was dispensable for immunity against low-dose *M. marinum* infection (Fig. 1C). Thus, we conclude that *pycard* is not essential for innate immune defence of larval zebrafish in a low-dose mycobacterial infection. This supports earlier findings from Matty et al. (2019), suggesting that the innate immune response against *M. marinum* infection in larval zebrafish is not dependent on Pycard.

To determine whether *pycard* deficiency affects bacterial burden, we infected two- to 1000-cell stage embryos originating from *pycard^+/tpu4^* F5 parents with a low dose of *M. marinum* (Fig. 1D). Bacterial copy number in 4 dpi (days post infection) larvae was similar in *pycard^tpu4/tpu4^* larvae compared to *pycard^+/tpu4^* and *pycard^+/+^* larvae [median colony-forming units (CFU): 32,805, 23,812 and 32,721, respectively] (Fig. 1D). To study further the role of *pycard* in the context of larval mycobacterial infection, we carried out bacterial burden analysis on *pycard^tpu4^* and *pycard^tpu5^* mutant lines using another infection model (Fig. 1E,F). At 2 dpf, larvae originating from *pycard^+/tpu4^* or *pycard^+/tpu5^* F6 parents were infected with *M. marinum* into blood circulation valley, and bacterial copy number was quantified at 5 dpi. When compared to WT or heterozygous larvae, there were no differences in bacterial copy numbers in *pycard^tpu4/tpu4^* or *pycard^tpu5/tpu5^* larvae (Fig. 1E,F). Thus, *pycard* expression is dispensable for the innate immune response against *M. marinum* in zebrafish larvae.

### *pycard* is essential for defence against *M. marinum* infection in adult zebrafish

**Fig. 2.**
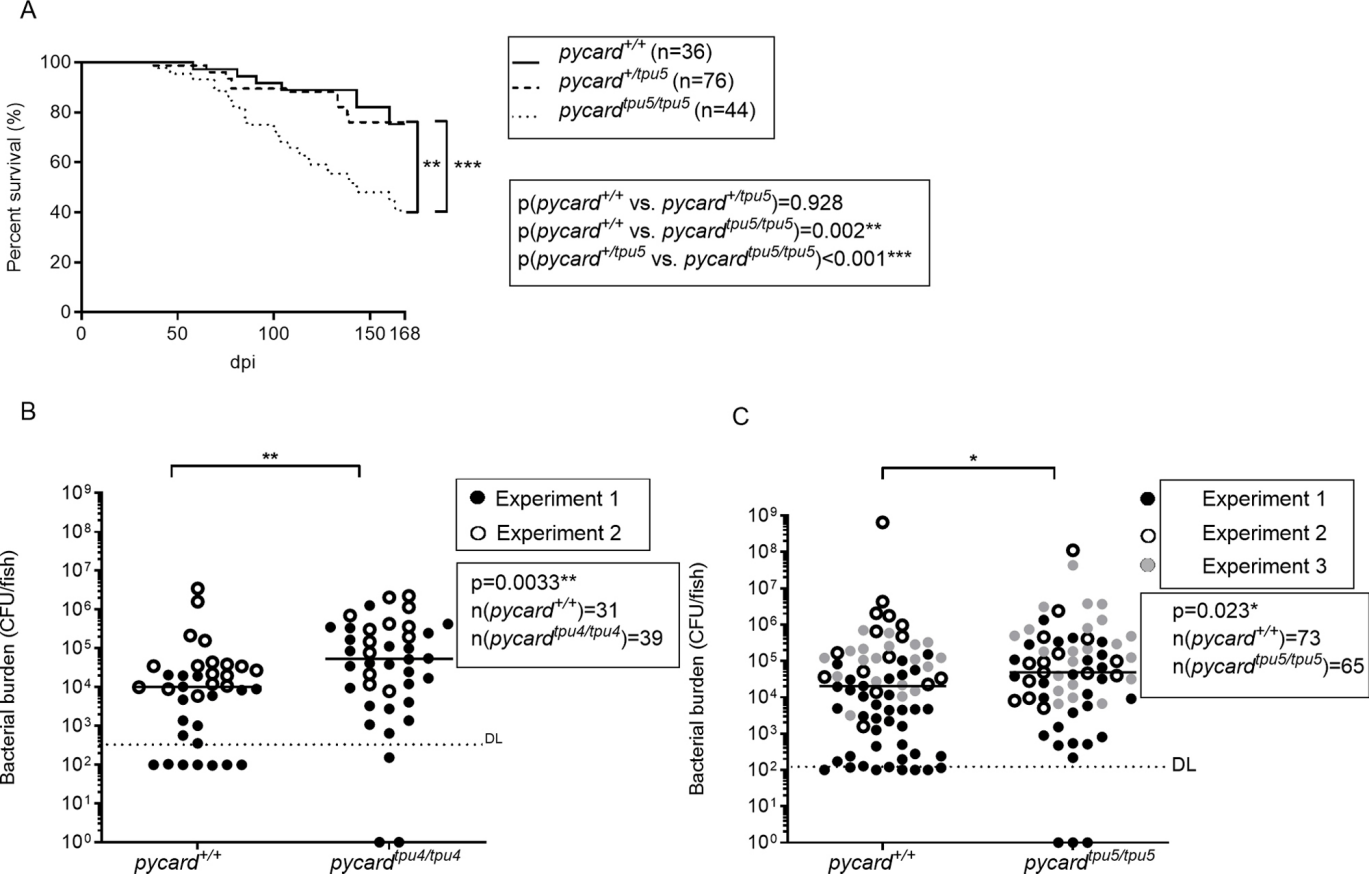
***pycard*^−/−^ adult fish display reduced survival and a higher bacterial burden upon mycobacterial infection.** (A) Adult fish of the *pycard^tpu5^* line were infected with a low dose of *M. marinum*, and their survival was followed daily. The fish were genotyped post-mortem. Survival data are presented as a Kaplan–Meier survival curve. Statistical analysis was performed with the log-rank test. The data have been pooled from two experiments. Individual experiments, with bacterial doses, are shown in Fig. S2. (B,C) Zebrafish from the mutant line *pycard^tpu4^* (medians: *pycard^+/+^*, 10,045 CFU; *pycard^tpu4/tpu4^*, 53,426 CFU; **P=0.0033) (B) and the mutant line *pycard^tpu5^* (medians: *pycard^+/+^*, 20,349 CFU; *pycard^tpu5/tpu5^*, 48,648 CFU; *P=0.023) (C). Fish were infected with a low dose of *M. marinum*, and the bacterial burden was analysed at 4 weeks post infection (wpi) from whole-organ block DNA using qPCR with *M. marinum* genome-specific primers. The experiment was performed twice for *pycard^tpu4^* and three times for *pycard^tpu5^*. Individual experiments are indicated by different symbols and are shown in separate graphs, with the bacterial dose and the respective statistics in Fig. S3. The line indicates the median. Data were analysed with the Mann–Whitney *U*-test, two tailed. For statistical purposes, samples in which the bacterial burden was below the detection limit, *pycard*^+/+^ fish were designated a value of 100 CFU and *pycard*^−/−^ fish a value of 0 CFU. Both sexes were included in the experiment in approximately equal numbers.

Inflammasomes are often regarded as a mechanism of innate immunity (Wu et al., 2024b). However, recent evidence points towards a role also in adaptive immunity (Deets and Vance, 2021). Moreover, Nlrp3 and Pycard might have inflammasome-independent roles (Javanmard Khameneh et al., 2019; McElvania Tekippe et al., 2010; Taxman et al., 2011; Yan et al., 2018)*.* To this end, we studied whether *pycard* affects immunity against *M. marinum* in adult zebrafish by following survival during a low-dose mycobacterial infection. The survival of the knockout *pycard^tpu5/tpu5^* fish was markedly impaired with low-dose infection, with end-point survival being 75.2% for *pycard^+/+^* and 40.6% for *pycard^tpu5/tpu5^* (**P=0.002) (Fig. 2A). To investigate whether reduced survival is attributed to compromised resistance or tolerance, we determined the bacterial burden of infected mutants and WT siblings. Adult zebrafish were infected with a low dose of *M. marinum*, and bacterial burden of the fish was measured at 4 weeks post infection (wpi) in two independent mutant lines for *pycard* (Fig. 2B,C). We found that the bacterial burden was significantly increased in both mutants in internal organ block [for *pycard^tpu4^*, medians were *pycard^+/+^*, 10,045 CFU; *pycard^tpu4/tpu4^*, 53,426 CFU (**P=0.0033); for *pycard^tpu5^*, medians were *pycard^+/+^*, 20,349 CFU; *pycard^tpu5/tpu5^*, 48,648 CFU (*P=0.023)] (Fig. 2B,C). These data indicate that *pycard* expression is required for normal resistance against a low-dose *M. marinum* infection in adult zebrafish. Results and bacterial doses from the individual experiments are shown in Figs S2 and S3.

### *pycard^tpu4/tpu4^* knockout adult fish have normal blood cell distribution

**Fig. 3.**
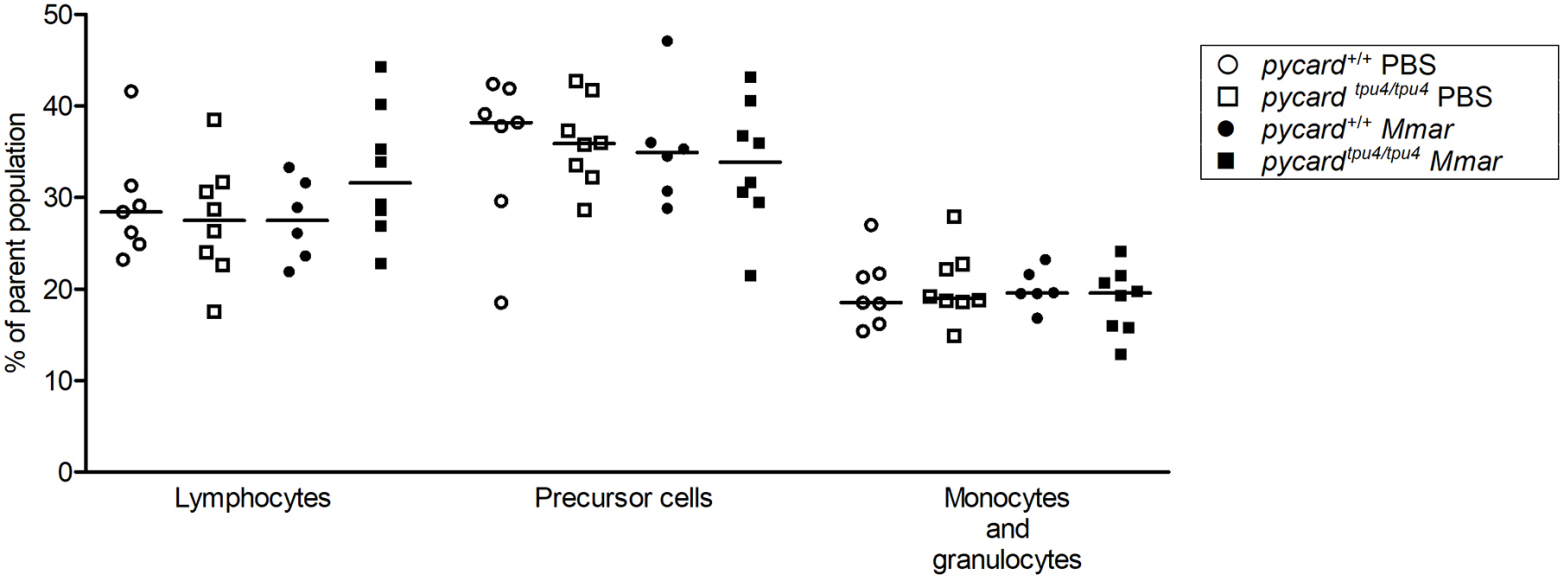
**Unchallenged as well as *M. marinum*-infected *pycard^tpu4/tpu4^* mutants have normal distribution of blood cell populations.** Adult zebrafish were either mock injected (PBS) or infected with a low dose (experiment 1: mean, 17 CFU; range, 12-28 CFU; experiment 2: mean, 19 CFU; range 16-21 CFU) of *M. marinum* (*Mmar*). The whole-kidney marrow was analysed at 4 wpi using flow cytometry. Results have been pooled from two experiments (*n*=3-5 in each group, per experiment). Each datapoint represents an individual fish. The line indicates the median. See Fig. S4 for the individual experiments, including exact sample size, bacterial dose and the gating strategy. Both sexes were included in the experiment in approximately equal numbers.

Previously, inflammasome components have been implicated in haematopoiesis and myelopoiesis (Frame et al., 2020; Tyrkalska et al., 2019). As *pycard* knockout fish had higher bacterial burden than that of their WT siblings (Fig. 2B,C), we investigated whether altered resistance was associated with changes in the number of leukocytes. Thus, we collected kidneys, which are the main site of haematopoiesis in fish, from both *M. marinum*-challenged and mock [phosphate-buffered saline (PBS)]-injected adult zebrafish. At 4 wpi, mutant fish presented similar numbers of precursor cells, monocytes, granulocytes and lymphocytes to WT siblings (Fig. 3). The experiment was done twice, and pooled data are presented in Fig. 3. As the number of cells in mutants remained similar to that in WT fish, it appeared that *pycard* does not affect leukocyte development in the adult zebrafish kidney. Individual experiments, including bacterial doses, and a full gating strategy are displayed in Fig. S4. Of note, this type of analysis cannot distinguish between different leukocyte sub-populations.

### *pycard* is widely expressed across tissues

**Fig. 4.**
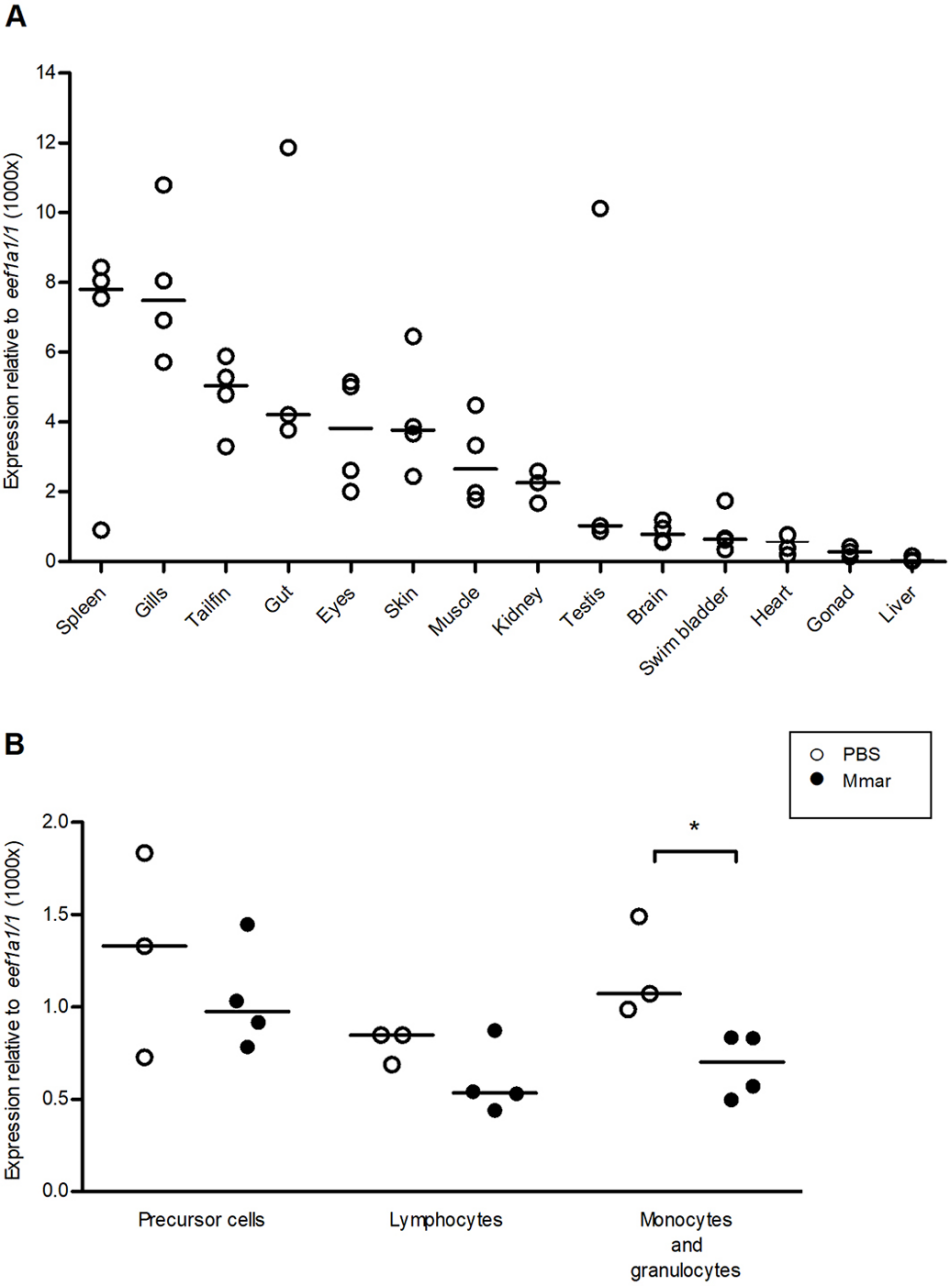
***pycard* is expressed in different tissues and blood cell types.** (A) Each datapoint represents the expression level of *pycard* in a single organ collected from a single AB fish (no treatment) [*n*=3 (gut, kidney, testis, gonad), *n*=4 (spleen, gills, tailfin, eyes, skin, muscle, brain, swim bladder, heart, liver)]. (B) AB zebrafish infected with *M. marinum* (Mmar) (mean, 28 CFU; range, 21-34 CFU) display a decrease in *pycard* expression in their monocyte and granulocyte population. Fish were either infected with *Mmar* or mock injected with PBS. At 4 wpi, the fish were euthanized and their kidneys were collected for fluorescence-activated cell sorting. RNA was extracted from the sorted populations, and *pycard* expression was measured with qPCR and normalized to *eef1a1/1* expression. Each sample contained kidneys from three fish, pooled (*n*=3-4). Both male and female fish were used in both experiments. The line indicates the median. *P(monocytes and granulocytes)=0.0286, Mann–Whitney, two tailed.

To gain further information on the role of *pycard* in zebrafish with and without *M. marinum* challenge, mRNA expression in tissues and fluorescence-activated cell sorting (FACS)-sorted blood cell samples of AB WT fish was analysed by qPCR. In uninfected tissues, highest median expression was seen in the spleen, gills, tailfin, gut, eyes, skin, muscle and kidney (Fig. 4A). The kidney and spleen are the main sites for haematopoiesis in zebrafish, whereas the gills, skin, tail, eyes and gut are immunologically significant as they are exposed to the surrounding environment. For blood cell analyses, fish were infected with a low dose of *M. marinum*, and samples were collected for FACS at 4 wpi. In the granulocyte population, which also harbours monocytes, *pycard* expression was decreased during the infection (Fig. 4B).

### Granulomas in *pycard^tpu/tpu4^* fish are larger than those in WT siblings

**Fig. 5.**
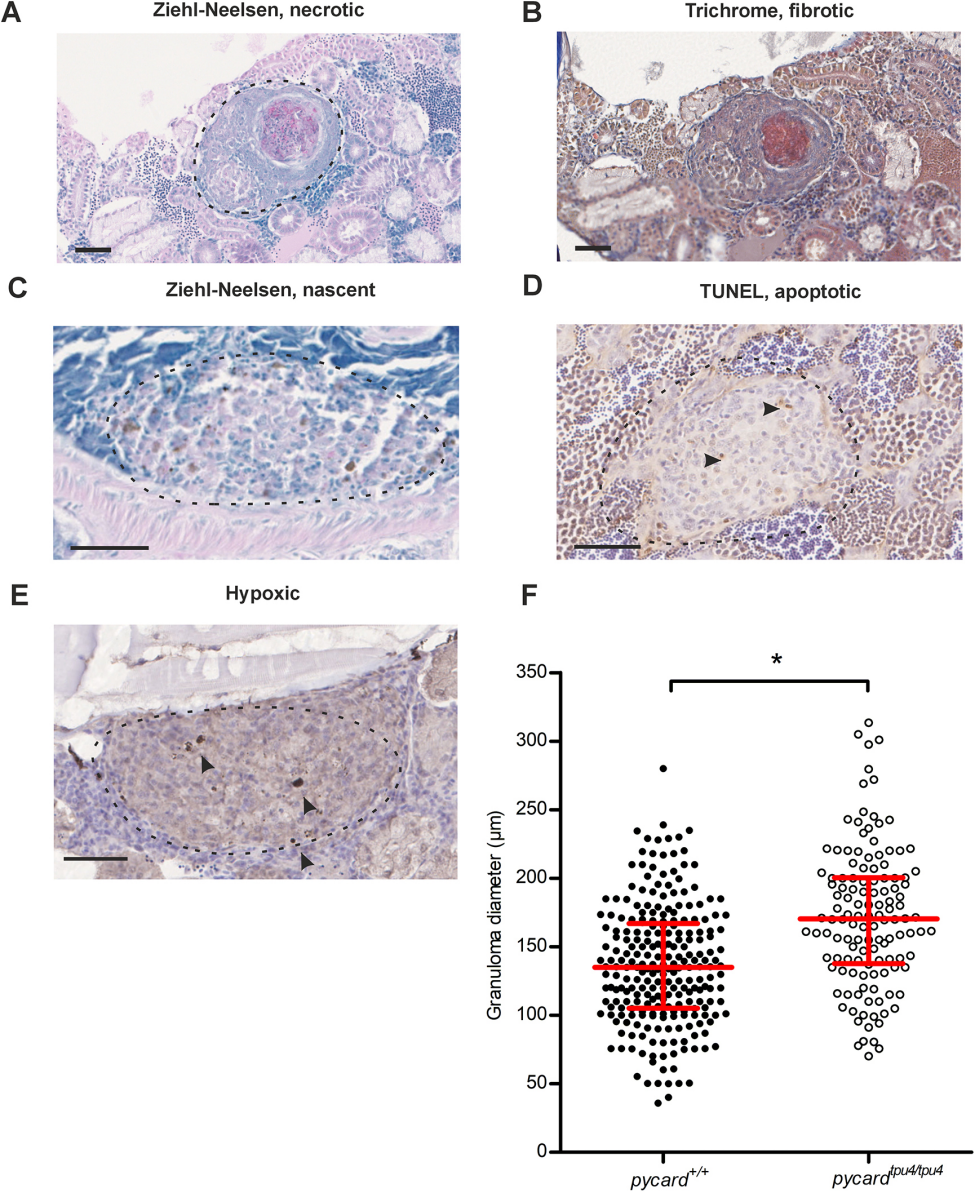
***pycard^tpu4/tpu4^* fish display an increase in granuloma size.** Six wild-type (WT) and four *pycard^tpu4/tpu4^* fish were infected with a low dose (mean, 64 CFU; range, 53-76 CFU) of *M. marinum*. At 8 wpi, the fish were processed for histological analysis of granulomas with Ziehl–Neelsen stain, Mallory's trichrome stain, hypoxia staining and TUNEL assay. (A) A necrotic and fibrotic granuloma stained with Ziehl–Neelsen stain. Dashed line indicates the area that was considered to belong to the capsule of the granuloma. (B) A necrotic and fibrotic granuloma stained with Mallory's trichrome stain. (C) A nascent granuloma, Ziehl–Neelsen staining. (D) A granuloma containing apoptotic cells, TUNEL assay. (E) A hypoxic granuloma. Scale bars: 50 µm. In B-E, dashed line indicates the borders of the granuloma; arrowheads indicate the positively stained cells. (F) The numbers and characteristics of each granuloma [*n*=244 (*pycard^+/+^*), *n*=128 (*pycard^tpu4/tpu4^*)] were recorded for each fish. Using a linear mixed model, granulomas in *pycard^tpu4/tpu4^* were determined to be larger than those in WT siblings (*P=0.0217) using R-package lme4, with fish as a random and genotype as a fixed factor. The line indicates the median and the interquartile range. Only male fish were used as female fish often present an increased number of small granulomas in the gonads, which complicates analyses. See also Fig. S5 for sizes of individual fish granulomas and Fig. S6 for characterization.

Adult zebrafish develop mycobacterial granulomas with caseous necrosis, hypoxic core and a fibrous cuff, but fewer lymphocytes than mammalian granulomas (Myllymäki et al., 2018; Parikka et al., 2012; Swaim et al., 2007). We next investigated whether lack of *pycard* expression affects granuloma formation in the adult zebrafish. The adult zebrafish were infected with a low dose (mean, 64 CFU; range, 53-76 CFU) of *M. marinum*, and granulomas were characterized from the histological sections using Ziehl–Neelsen, Mallory's trichrome or hypoxia staining, or terminal deoxynucleotidyl transferase dUTP nick-end labelling (TUNEL) assay (Fig. 5A-E) of samples collected at 8 wpi. As shown in Fig. 5F, *pycard^tpu4/tpu4^* adult fish had larger granulomas than those of WT siblings (mean for *pycard^+/+^*, 140.8 µm; mean for *pycard^tpu4/tpu4^*, 167.1 µm; *P=0.0217). We also analysed the type of granuloma in mutant and WT fish. Classification to nascent, necrotic and hypoxic granulomas was done as described in Myllymäki et al. (2018). We also analysed, with Mallory's trichrome staining, whether granulomas had fibrotic capsule (Fig. 5B) and, with TUNEL assay (Fig. 5D), whether there were apoptotic cells inside granulomas. When granuloma size was analysed for each granuloma type separately, *pycard^tpu4/tpu4^* fish had larger nascent, fibrous, necrotic and apoptotic granulomas compared to those of WT fish (Fig. S5B-F). However, there were no notable differences in the distribution of different types of granulomas in *pycard^tpu4/tpu4^* compared to *pycard^+/+^* fish (Fig. S6). These data suggest that *pycard^tpu4/tpu4^* fish are less capable of containing bacterial growth within the granulomas, leading to increased bacterial burden and, subsequently, to compromised immunity against *M. marinum*.

### Pycard affects the expression of haematopoietic transcription factors in *M. marinum* infection

**Fig. 6.**
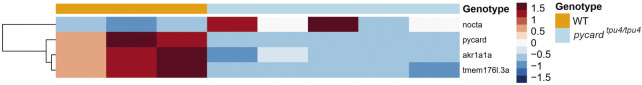
**Heatmap of the RNA-seq results in *pycard^tpu4/tpu4^* adult zebrafish mock injected with PBS.** Adult zebrafish from the *pycard^tpu4^* mutant line were mock injected and at 4[Supplementary-material sup1]wpi sacrificed for analysis. Whole-kidney marrow was used for the RNA-seq analysis of male fish [*n*(WT)=3, *n*(*pycard^tpu4/tpu4^*)=5]. See also Table S1. Expressions are centred and scaled by row. Hierarchical clustering with Euclidean distance and complete linkage method was used to order genes.

**Fig. 7.**
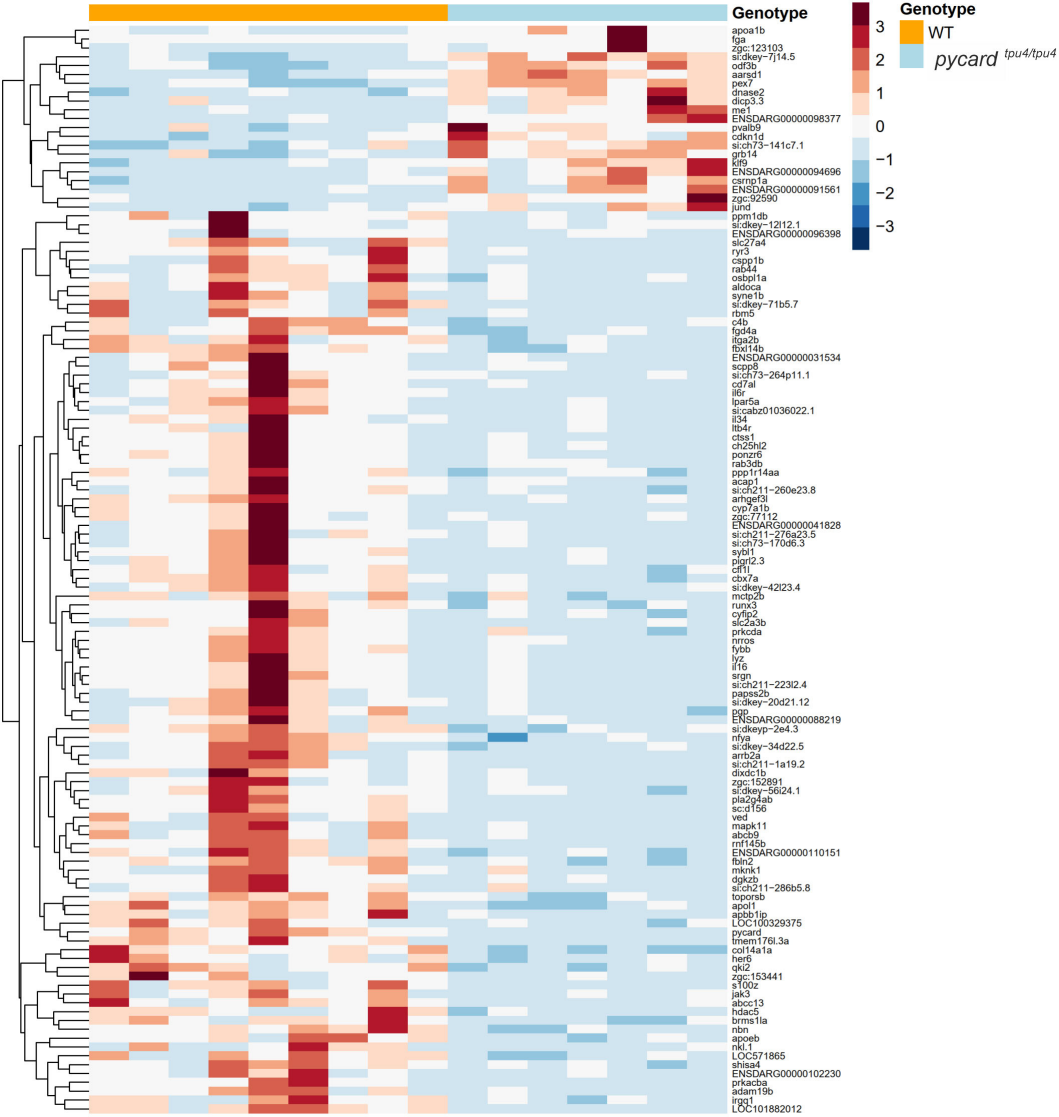
**Heatmap of the RNA-seq results in adult *pycard^tpu4/tpu4^* zebrafish infected with a low dose of *M. marinum.*** Adult zebrafish from the *pycard^tpu4^* mutant line were infected with a low dose of *M. marinum* (mean dose, 31 CFU; range, 16-48 CFU), and, at 4[Supplementary-material sup1]wpi, they were sacrificed for analysis. Whole-kidney marrow was used for RNA-seq analysis in male fish [*n*(WT)=9, *n*(*pycard^tpu4/tpu4^*)=7]. See also Tables S2 and S3. Expressions are centred and scaled by row. Hierarchical clustering with Euclidean distance and complete linkage method was used to order genes.

To identify the cause of the increased susceptibility of *pycard^−/−^* zebrafish to *M. marinum* infection, transcriptomes of *pycard^tpu4^* mutants, as well as WT controls, from the kidney marrow at 4 wpi after a low dose of *M. marinum* (mean dose, 31 CFU; range, 16-48 CFU) or mock (PBS) injection were analysed with RNA-seq. Based on RNA-seq, four genes were differentially expressed [DESeq2 (Anders and Huber, 2010; Love et al., 2014), |log2 fold change| >1 between the groups, adjusted *P*-value <0.05] without mycobacterial challenge (Fig. 6; Table S1). In turn, infected mutants showed 123 differentially expressed genes, in comparison to WT siblings, 21 of which were upregulated and 102 downregulated, in the *pycard^tpu4^* mutant compared to WT (Fig. 7; Tables S2 and S3).

We observed that a neutrophil marker, *lysozyme* (*lyz*) was downregulated in *pycard* mutants. In addition, other cell type-specific genes, such as *cd7 antigen-like* (*cd7al*) (T-cell) (Carmona et al., 2017), *Janus kinase 3 (a protein tyrosine kinase, leukocyte)* (*jak3*) [natural killer (NK) cell], *integrin, alpha 2b* (*itga2b*) (thrombocyte) and *apolipoprotein Eb* (*apoeb*) (macrophages/myeloid cells) were among the downregulated genes [if not otherwise mentioned, classified to cell types according to the single-cell RNA-seq (scRNA-seq) by Tang et al. (2017), accessed via the online tool developed by Lareau et al. (2017)].

Next, we looked at whether the RNA-seq analysis gave indications for differences in immune-related gene expression. We observed that differentially expressed genes included several transcription factors, many of which are known to affect myelopoiesis in zebrafish, mouse or human. These include *ring finger protein 145b* (*rnf145b*) (Gieger et al., 2011), *Kruppel-like factor 9* (*klf9*) (Zhang et al., 2017), *chromobox homolog 7a* (*cbx7a*) (Jung et al., 2019), *nuclear transcription factor Y, alpha* (*nfya*) (Zhu et al., 2005), *histone deacetylase 5* (*hdac5*) (Watamoto et al., 2003), *RUNX family transcription factor 3* (*runx3*) (Kalev-Zylinska et al., 2003) and *cysteine-serine-rich nuclear protein 1a* (*csrnp1a*) (Espina et al., 2013). Based on the scRNA-seq data by Tang et al. (2017), accessed via the online tool developed by Lareau et al. (2017), the majority of the downregulated genes (60 out of 102) in the *pycard^tpu4/tpu4^* fish are expressed in neutrophils (Table S3). The upregulated genes do not present such a definite pattern (Table S2). We also divided the differently expressed genes into functional categories (Tables S1-S3).

After the RNA-seq analysis, we used the other mutant line, *pycard^tpu5^*, to validate the results. To this end, we performed either a mock injection (PBS) or a low-dose infection (mean, 24 CFU; range, 16-32 CFU) to *pycard^tpu5/tpu5^* and WT siblings and analysed the expression of ten selected genes in kidney tissue at 4 wpi with qPCR. The results for *pycard* itself, for a putative negative regulator of inflammasome, namely *transmembrane protein 176l.3a* (*tmem176l.3a*) (Segovia et al., 2019), for a potentially a T cell-activating gene, *diverse immunoglobulin domain-containing protein 3.3* (*dicp3.3*), and for *malic enzyme 1* (*me1*) were replicated, whereas the results for *aldo-keto reductase family 1, member A1a (aldehyde reductase)* (*akr1a1a*), *klf9*, *itga2b*, *lyz*, *nocturnin a* (*nocta*) and *cbx7a* did not differ between *pycard^tpu5^* mutants and controls (Fig. S7). *tmem176l.3a* was differentially expressed after mock injection, whereas *dicp3.3* and *me1* were differently expressed after *M. marinum* infection.

RNA-seq data suggest a role for *pycard* in the demand-adapted response of immune cells. To study the role of Pycard in myelopoiesis, we crossed homozygous *pycard^tpu4^* to transgenic zebrafish lines, *Tg(mpx:GFP)i114 (AB)* and *Tg(mpeg1.1:GFP)ka101 (AB)*, with fluorescent neutrophils and macrophages, respectively. Thus, we obtained zebrafish that are heterozygous for the mutation and carry fluorescent neutrophils or macrophages. We incrossed these fish to obtain homozygous, heterozygous and WT larvae for *pycard^tpu4^*. At 3 dpf, we wounded the tail fin with a 30 G needle in order to trigger demand-driven haematopoiesis, and imaged fish to quantify the number of neutrophils and macrophages. In this setting, we saw no difference in the number of these cells between the WT and the heterozygous or homozygous mutants (Fig. S8A,B).

To study the effect of *pycard* defect on myelopoiesis during *M. marinum* infection, we infected *pycard^tpu4^* and *pycard^tpu5^* crossed to *Tg(mpx:GFP)i114 (AB)* or *Tg(mpeg1.1:GFP)ka101 (AB)* at 2 dpf with *M. marinum* (mean dose, 180 CFU; range, 97-330 CFU) into blood circulation valley. At 1 dpi and 2 dpi, we imaged larvae to quantify the number of neutrophils and macrophages. At both timepoints, we saw no difference in the cell counts between *pycard* mutants and WT larvae (Fig. S8C,D). As anticipated, the number of neutrophils was elevated at both time points in all of the groups (Fig. S8C), indicating demand-driven myelopoiesis.

### The effect of Pycard on the transcriptomic profile of neutrophils during *M. marinum* infection

**Fig. 8.**
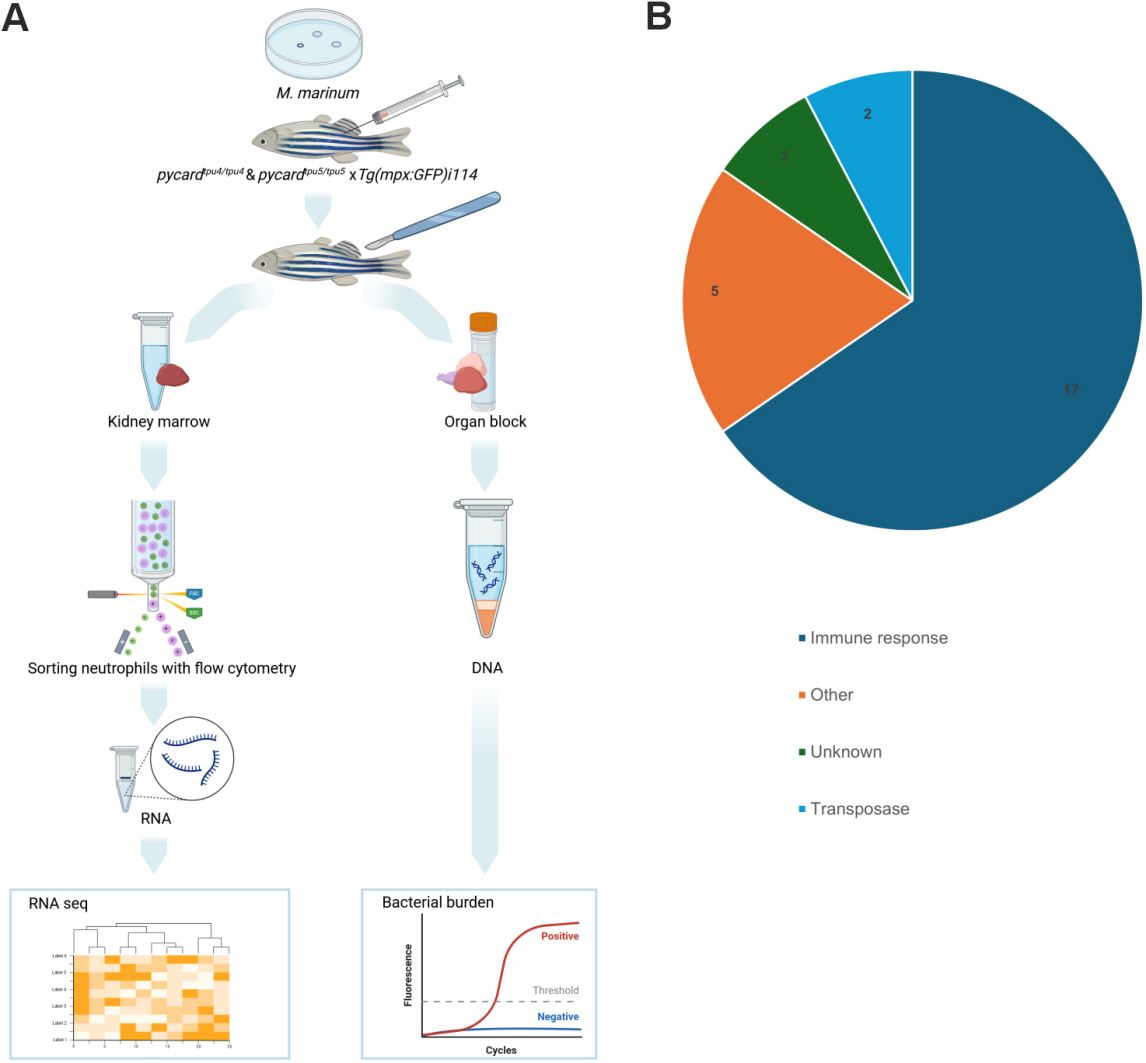
**Transcriptomic analysis of kidney marrow-derived neutrophils and categorization of differentially expressed genes in adult *pycard^tpu4/tpu4^* and *pycard^tpu5/tpu5^* zebrafish infected with *M. marinum*.** (A) Fish homozygous for *pycard^tpu4^* and *pycard^tpu5^*, and WT controls crossed to *Tg(mpx:GFP)i114 (AB)* were infected with *M. marinum* [mean dose, 95 CFU; range, 69-118 CFU (*pycard^tpu4^*); mean dose, 6 CFU; range, 4-8 CFU (*pycard^tpu5^*)]. At 4 wpi, fish were euthanized and dissected to obtain a kidney marrow and a whole-organ block. From a suspended kidney marrow, fluorescent (GFP) neutrophils were sorted with flow cytometry, and RNA was extracted for transcriptomic analysis with RNA-seq [*n*(WT)=9, (*pycard^tpu4/tpu4^*)=3, (*pycard^tpu5/tpu5^*)=6]. The experiment was performed once for *pycard^tpu4^* and *pycard^tpu5^* fish. Both sexes were included in the experiment. To monitor the effect of varying bacterial burden on transcriptomics, bacterial copy number was determined with qPCR from DNA extracted from the whole-organ block. Created in BioRender by Junno, M. (2025). https://BioRender.com/d75w516. This figure was sublicensed under CC-BY 4.0 terms. (B) Differentially expressed genes were categorized according to the literature (Table S4).

To study in more detail the effect of *pycard* deficiency on neutrophil in *M. marinum* infection, fish homozygous for *pycard^tpu4^* and *pycard^tpu5^* [*Tg(mpx:GFP)i114 (AB)*], as well as WT controls, both having fluorescently marked neutrophils, were infected with *M. marinum* [mean dose, 95 CFU; range, 69-118 CFU (*pycard^tpu4^*); mean dose, 6 CFU; range 4-8 CFU (*pycard^tpu5^*)] and kidney marrow-derived neutrophils were sorted with FACS at 4 wpi (Fig. S9A,B). There was no statistically significant difference in the relative neutrophil count between the WT controls and homozygous *pycard^tpu4^* and *pycard^tpu5^* fish (median, 75.1% versus 71.2%; Fig. S9B). Transcriptome of the sorted neutrophils was analysed with RNA-seq (Fig. 8A). To verify the infection, whole organ blocks were collected, and bacterial burden was quantified (Fig. S9C). As shown in Fig. S9D,E, the 20 most expressed genes in WT fish are dominantly expressed in neutrophils [according to the data by Tang et al. (2017)]. This indicated that FACS-based sorting of GFP-expressing cells had yielded neutrophils.

According to the RNA-seq results, *pycard^tpu4/tpu4^* and *pycard^tpu5/tpu5^* zebrafish shared 26 differentially expressed genes [DESeq2 (Anders and Huber, 2010; Love et al., 2014), |log2 fold change|>1 and |log2 fold change of medians|>1 between the groups during *M. marinum* infection] (Table S4). In addition to *pycard*, 18 genes were downregulated. Most of these genes have a defined role in immune response (Fig. 8B, Table S4). Differentially expressed genes included, for example, *interleukin 11 receptor, alpha* (*il11ra*) (Metcalfe et al., 2020), *SWI/SNF related, matrix associated, actin dependent regulator of chromatin, subfamily c, member 1a* (*smarccia1a*) (Liu et al., 2023), *deoxyribonuclease II, lysosomal* (*dnase2*) (Hong et al., 2020; Rodero et al., 2017; Fujihara et al., 2016), *ferritin, heavy polypeptide-like 28* (*fthl28*) (Dai et al., 2023; Liu et al., 2010) and *si:ch211-135n15.2* (Blum et al., 2025). Among seven upregulated genes, we observed *S100 calcium binding protein A10b* (*s100a10b*), identified as a negative regulator of Toll-like receptor signalling (Lou et al., 2020). In total, 17 out of 26 differentially expressed genes or their orthologs were related to immune response (Fig. 8B; Table S4). The detailed list of the genes can be found in Table S4.

## DISCUSSION

In this study, we used CRISPR-Cas9 to produce two knockout zebrafish lines, devoid of the inflammasome adaptor gene *pycard* and studied their phenotype during *M. marinum* infection. qPCR analysis of the larvae showed that both *pycard* knockout lines (*pycard^tpu4^* and *pycard^tpu5^*) present diminished *pycard* transcript levels (Fig. S1). Moreover, infected *pycard^tpu4/tpu4^* or *pycard^tpu5/tpu5^* larval zebrafish did not present altered survival or bacterial burden in *M. marinum* infection, indicating that *pycard* is dispensable for innate immune response against *M. marinum* in zebrafish larvae (Fig. 1). In turn, analyses of the adult zebrafish revealed increased susceptibility of the *pycard^tpu5/tpu5^* fish to mycobacterial infection. In addition, the bacterial burden was increased in both *pycard* mutant lines (*pycard^tpu4^* and *pycard^tpu5^*) (Fig. 2)*.* Altered resistance was accompanied by changes in transcription, especially in the genes related to neutrophil function (Fig. 7; Table S3). A previous zebrafish study (Matty et al., 2019) showed that the survival and bacterial burden of *pycard* knockout larvae were not markedly affected in *M. marinum* infection. Our data indicate that the role of *pycard* is more important in mycobacterial immunity in adult zebrafish than in larvae. This might be due to differences in the immune response but also to very different infection kinetics between these two models. However, we consider the adult model informative as it includes both the innate and adaptive immune responses and therefore reflects better mammalian models of tuberculosis.

The inflammasome adaptor protein-coding gene *pycard* is required for inflammasome signalling and IL-1β production in zebrafish (Li et al., 2018). In mouse, the lack of Pycard affects the course of mycobacterial infection (Mayer-Barber et al., 2010; McElvania Tekippe et al., 2010). In *NLR family, CARD domain containing 3-like* (*nlrc3l*)-deficient zebrafish embryos, uncontrolled Pycard-dependent inflammasome activation led to increased immune cell infiltration and tissue damage during *M. marinum* infection (Niu et al., 2022). In addition, PYCARD has been suggested to regulate the immune response independent of the inflammasome (Cheong et al., 2020; Javanmard Khameneh et al., 2019; Taxman et al., 2006; Yan et al., 2018). In two studies, in a mouse model, Pycard-deficient mice have reduced survival against Mtb H37Rv infection (Mayer-Barber et al., 2010; McElvania Tekippe et al., 2010). There were some differences in the phenotypes between these studies, however. Whereas McElvania Tekippe et al. (2010) found that survival from Mtb H37Rv strain infection was dependent on Pycard but not caspase 1, Mayer-Barber et al. (2010) observed a decreased survival in caspase 1-deficient mice. There is no obvious explanation for this discrepancy, although it could be a result of the different infection procedure used in the two studies [Mayer-Barber et al. (2010), 50-100 CFU; McElvania Tekippe et al. (2010), 250-350 CFU]. In addition, McElvania Tekippe et al. (2010) found that, in Pycard-deficient mice, a number of bacteria are found outside the granulomas owing to an inability to contain the mycobacteria in the granulomas. In our study, *pycard^tpu4/tpu4^* adult zebrafish presented larger granulomas than those of their WT siblings (Fig. 5). This could be due to, for example, the granulomas being less able to contain the bacteria, leading to infection progressing faster, which would be in line with higher bacterial burden as well as earlier death in the adult fish.

Based on our RNA-seq analysis and further independent validation with qPCR, there were few genes for which expression was affected by the loss of *pycard* in the kidney marrow of unchallenged adult zebrafish. In addition to the expression of *pycard* itself, the expression of *tmem176l.3a* was downregulated. Of note, the mouse ortholog *Tmem176b* has been shown to be a negative regulator of inflammasome signalling (Segovia et al., 2019). *tmem176l.3a* was also downregulated after *M. marinum* infection in kidneys and neutrophils of the *pycard*-deficient fish.

During *M. marinum* infection, over half of the (60 out of 102) downregulated genes in our data on the differentially expressed genes are expressed in neutrophils, according to the scRNA-seq data by Tang et al. (2017) (Table S3). Neutrophils are known to play a dual role in tuberculosis in mouse models (Pedrosa et al., 2000; Sugawara et al., 2004). As neutrophils are the most numerous immune cell type in blood, they are often among the first responders to an infection and thus shape the initial response by initiating cytokine signalling. However, in an active infection, prolonged neutrophil activation can cause damage in host tissues [reviewed by Kroon et al. (2018)]. Using zebrafish larvae, Kenyon et al. (2017) found many inflammasome-related genes upregulated in neutrophils at the early stage of an *M. marinum* infection in zebrafish larvae.

Inflammasomes have been shown to regulate haematopoiesis in zebrafish, mice and humans (Frame et al., 2020; Tyrkalska et al., 2019; Zhao et al., 2014). Based on studies employing morpholino silencing of *pycard* in zebrafish larvae, it was suggested that inflammasome cleaves the main haematopoietic transcription factor GATA binding protein 1a (Gata1a) into an active state (Tyrkalska et al., 2019). According to Tyrkalska et al. (2019), Gata1a, in combination with Spi-1 proto-oncogene b (Spi1b), is responsible for the decreased numbers of macrophages and neutrophils in zebrafish larvae in demand-driven haematopoiesis in morphant zebrafish. Frame et al. (2020) showed, in a similar zebrafish model for *pycard* silencing, that inflammasome stimulation increased multilineage hematopoietic colony-forming units and T-cell progenitors. They also showed an increase in *interleukin 6* (*il6*) expression after exogenous *interleukin 1, beta* (*il1b*) induction by glucose, a signal that has been associated with promoting haematopoietic stem cell differentiation (Frame et al., 2020; Zhao et al., 2014). Supporting this, our kidney RNA-seq data show downregulation of *interleukin 6 receptor* (*il6r*) in *pycard^tpu4/tpu4^* mutants after *M. marinum* challenge.

Expression analysis of kidney samples in our study suggests compromised haematopoiesis upon mycobacterial challenge in knockout fish (*pycard*^−/−^). These include reduced expression of myelopoiesis- and haematopoiesis-associated transcription factors *rnf145b* (Gieger et al., 2011), *nfya* (Zhu et al., 2005), *hdac5* (Watamoto et al., 2003) and *runx3* (Kalev-Zylinska et al., 2003), and elevated expression of *csrnp1a* (Espina et al., 2013). However, we are unable to determine in which specific cell types these transcription factors are expressed in our kidney samples. Furthermore, in our transcriptome analysis of the *pycard*-deficient neutrophils, the most downregulated gene in both *pycard^tpu4/tpu4^* and *pycard^tpu5/tpu5^* neutrophils was immunity-associated *si:ch211-135n15.2* expressed in hematopoietic cells (Blum et al., 2025).

Tyrkalska et al. (2019) showed decreased numbers of neutrophils and macrophages in unchallenged *pycard* morphants. However, according to Lozano-Gil et al. (2022), myeloid inflammasome does not affect haematopoiesis. In our setting, we could not see differences in neutrophil or macrophage counts in *pycard* mutant larvae post wounding or during *M. marinum* infection, perhaps due to maternal RNA or other compensatory mechanisms. The presence of impaired haematopoiesis was also not observed in the neutrophil counts, determined with FACS, from infected fish. This effect can be dependent on the progression of infection and would require further analysis. However, these results are supported by the fact that the neutrophil marker *mpx* used as a marker in our experiments did not show decreased expression owing to *pycard* deficiency in our kidney or neutrophil RNA-seq data.

Several genes that were differentially expressed in infected *pycard^tpu4/tpu4^* fish compared to WT controls have been associated with mycobacterial infection. Oxidative stress and reactive oxygen species (ROS) are known to be critical for host defence in tuberculosis, and ROS have also been suggested to regulate inflammasome activation in mice in an acute lung injury model (Cirillo et al., 2009; Kim et al., 2015). Genes associated with ROS formation were *me1*, which was also confirmed with qPCR with the other mutant line *pycard^tpu5/tpu5^*, and *negative regulator of reactive oxygen species* (*nrros*) (Lu et al., 2018; Noubade et al., 2014). We consider downregulation of *nrros* as a compensatory effect for compromised resistance. *interleukin 34* (*il34*) was also downregulated in our kidney RNA-seq data and is essential in macrophage migration in zebrafish (Wu et al., 2018). Moreover, IL34-stimulated macrophages were shown to be more resistant to mycobacterial entry and more efficient in phagolysosomal trafficking of *M. marinum* in *Xenopus laevis* (Popovic et al., 2019). Zebrafish Leukotriene B4 (Ltb4) activation mediates increased susceptibility to mycobacterial infection, and the Ltb4-inactivating enzyme Ltb4dh (also known as Ptgr1.1) counteracts this increase (Tobin et al., 2010, 2013). In Tobin et al. (2013), inhibiting the Leukotriene B4 receptor (Ltb4r) rescued the susceptible phenotype of Ltb4dh-deficient zebrafish (Tobin et al., 2013). *ltb4r* was downregulated in the *pycard^tpu4/tpu4^* mutants in our RNA-seq analysis. In addition, we observed significant downregulation of *NK-lysin 1* (*nkl.1*). Nkl.1 belongs to a family of proteins that have been indicated in the elimination of *M. tuberculosis*, although the direct homology of *nkl.1* and human granulysin remains to be shown (Stenger et al., 1999). In addition, we observed notable dysregulation of one gene associated with platelet activation: we observed an increase in the expression level of *fibrinogen alpha* (*fga*). Recently, Hortle et al. (2019) showed that platelet activation during infection compromises the host immunity in a mycobacterial infection in larval zebrafish. In their study, *fga* knockout larval zebrafish fish presented with a decreased bacterial burden (Hortle et al., 2019). Correspondingly *fga* was upregulated in our data. Platelets are known to be important for the immunopathology of tuberculosis, as they affect other immune cells, especially monocytes, to increase activation and enhance phagocytosis (Fox et al., 2018; Kirwan et al., 2021). These data suggest that *pycard^−/−^* zebrafish present a number of changes in their transcriptome, indicating a compromised host protective immune response in mycobacterial infection.

Transcriptomic analysis of *pycard^tpu4/tpu4^* and *pycard^tpu5/tpu5^* neutrophils performed in this article indicates a role for inflammasome activation in neutrophils during mycobacterial infection. For example, *fthl28* was downregulated in the neutrophils of *pycard^tpu4/tpu4^* and *pycard^tpu5/tpu5^* zebrafish. An ortholog of zebrafish *fthl28* has been associated with iron metabolism and immune response (Liu et al., 2010). Ferritin stimulates inflammasome activation (Fernandez-Rojo et al., 2024; Mehta et al., 2022) and induces neutrophil extracellular trap formation (Zhang et al., 2024; Jia et al., 2022). In addition, ferritin regulates iron homeostasis in macrophages, and *M. tuberculosis* can exploit host iron metabolism for survival (Dai et al., 2023). *dnase2*, *smarcc1a* and *il11ra* were also downregulated in *pycard*-deficient neutrophils. Defects in DNases lead to accumulation of neutrophil extracellular networks (Angeletti et al., 2021), and defects in DNase II are connected to autoinflammation (Angeletti et al., 2021; Hong et al., 2020; Rodero et al., 2017). *Smarcc1* activates inflammatory genes (Liu et al., 2023) and contributes to hematopoietic regeneration (Wu et al., 2024a). Il-11 inhibits pro-inflammatory cytokine release via receptor complex containing Il-11ra subunit (Ritter et al., 2020) and contributes to neutrophil recruitment during pulmonary infection (Traber et al., 2019). In genetically susceptible mice, Il-11 levels correlated with the severity of *M. tuberculosis* infection (Kapina et al., 2011).

Our results propose a role for *pycard* in defence against mycobacterial infection through regulation of a number of haematopoiesis- and myelopoiesis-associated genes and genes associated with neutrophil defence. Whether the dysregulated genes directly or indirectly associate with *pycard* remains to be studied.

We show that *pycard* expression is required for normal immunity against *M. marinum* in zebrafish. Our data highlight the difference between larval and adult zebrafish models. We show that *pycard* mutants form granulomas in a similar manner to WT control siblings. However, granulomas of the *pycard^tpu4/tpu4^* fish are larger than those of *pycard*^+/+^ fish, suggesting that *pycard* mutants have compromised ability to restrict bacterial growth in granulomas. There are several transcriptional changes in mutants linking *pycard* to neutrophil function. These gene findings suggest potential implications for mycobacterial resistance, but further research is needed to confirm their biological significance.

## MATERIALS AND METHODS

### Zebrafish lines and maintenance

All maintenance and experiments were done in accordance with the Finnish act on the protection of animals used for scientific or educational purposes (497/2013) and the EU Directive on the protection of animals used for scientific purposes (2010/63/EU). Permits for experiments were applied from the Regional State administrative agency (ESAVI/10079/04.10.06/2015 ESAVI/11144/04.10.07/2017, ESAVI/2776/2019, ESAVI/7251/2021 and ESAVI/12569/2024). Before infection experiments, adult fish were maintained in a flow-through system (Aquatic Habitats, Apopka, FL, USA). Fish were fed once a day with suitable granularity of Gemma Micro feed (Planktovie, Marseille, France). The light/dark cycle was 14 h/10 h in all laboratories and incubators. Embryos were kept in embryonic medium (5 mM NaCl, 0.17 mM KCl, 0.33 mM CaCl_2_, 0.33 mM MgSO_4_, 0.00001% Methylene Blue) in a 28.5°C incubator until 6 dpf and fed starting from 5 dpf.

Infected adult fish were kept in a separate laboratory in another flow through system (Aqua Schwartz mbH, Gönningen, Germany). Infected embryos were kept in 24-well plates in individual wells to prevent the spread of the infection. During infections, the wellbeing of the fish was followed at least once a day, and fish exhibiting symptoms, signs of pain or discomfort were euthanized with an overdose of a Tricaine anaesthetic [ethyl 3-aminobenzoate methanesulfonate (Merck, Kenilworth, NJ, USA)]. Adult fish used for experiments were 3-16 months of age and of both sexes. Fish lines used were AB WT or CRISPR-Cas9 mutants generated in house. CRISPR-Cas9 mutated fish were outcrossed to TL WT (*Tüpfel long fin, gja5b^t1/t1^, lof^dt2/dt2^*), so their background was AB×TL. All fish used in the experiments were the offspring of heterozygous parents, except the larvae used in the F3 survival experiment, as indicated in the text. The lines were assigned the Zebrafish Information Network (ZFIN) identifiers *pycard^tpu4^* and *pycard^tpu5^*. Each result indicates which line had been used for the experiment. The transgenic zebrafish lines *Tg(mpx:GFP)i114 (AB)* (Renshaw et al., 2006) and *Tg(mpeg1.1:GFP)ka101 (AB)*, used to produce *pycard* mutants with fluorescent neutrophils or macrophages, respectively, were obtained from the European Zebrafish Resource Center (EZRC; Karlsruhe Institute of Technology, Eggenstein-Leopoldshafen, Germany).

### CRISPR-Cas9 mutagenesis and genotyping

The design procedure of our in house-produced CRISPR-Cas9 mutant lines has been described in Ojanen et al. (2019). Briefly, several online tools were used for selecting several guide RNA (gRNA) targets for *pycard* (ENSDARG00000040076.8). The gRNA was synthesized with the MEGAShortScript T7 transcription kit (Thermo Fisher Scientific, Waltham, MA, USA). The in house-produced Cas9 protein was obtained from the Tampere Facility for protein services. Injections were performed with borosilicate capillary needles generated with Flaming/Brown micropipette puller (Sutter, Novato, CA, USA), using a micro injector (PV830 Pneumatic PicoPump, World Precision Instruments, Sarasota, FL, USA). For these, 130 pg gRNA and 250 pg Cas9 protein were co-injected into one-cell stage fertilized embryos of AB WT fish with Phenol Red (114537-5G, Merck) as a tracer dye. The CRISPR-injected F0 generation was crossed with TL WT fish, and, from this F1 generation, founder fish carrying frameshift-causing mutations were identified with a heteroduplex mobility assay and then by Sanger sequencing. After identification of the desirable mutations, the F1 heterozygous mutant fish were incrossed to obtain generation F2 (25% WT, 50% heterozygous mutants, 25% homozygous mutants). F3 and F4 generations were used for larval experiments where the effect of the functional maternal *pycard* mRNA could be excluded. In bacterial burden analysis of larvae, F6 and F7 generations were used.

Fish used for experiments were genotyped by assessing the restriction fragment length with agarose gel electrophoresis, after digestion of a PCR amplicon with the CseI restriction enzyme (Thermo Fisher Scientific). DNA extractions for genotyping were always done from whole larvae or from adult tailfins excised under anaesthesia. Briefly, samples were lysed with a standard lysis buffer [10 mM Tris-HCl (pH 8.2), 10 mM EDTA, 200 mM NaCl, 0.5% SDS] with Proteinase K (Thermo Fisher Scientific) (0.2 mg/ml) for a minimum of 2 h in a 55°C water bath. For larval bacterial burden analysis, a fixed lysis time of 21.5 h was used. Two volumes of absolute ethanol were added, and DNA was precipitated at −20°C for minimum of 30 min. The DNA was then pelleted for 20 min at 16,000 ***g*** in a microcentrifuge. The pellet was washed with 200 µl of 70% ethanol before suspending it in nuclease-free water. PCR was done with Dream Taq Hot Start polymerase (Thermo Fisher Scientific) with gene-specific primers (F, 5′-GACCCAACTGTGAGGAACCATG-3′; R, 5′-GCTTTCTTCAGACTTAAACGCCTTC-3′). Digested fragments were separated on a 2% (w/v) agarose gel using electrophoresis.

### Adult fish experiments

Offspring of a heterozygous fish crossing were used for the survival and bacterial burden assays, and fish were injected without knowing the genotypes beforehand, with the exception of fish injected for RNA-seq analysis of neutrophils. Fish were infected with a low dose of *M. marinum* intraperitoneally, under 0.02% Tricaine anaesthesia. The wellbeing of the fish was followed a minimum of once per day. Fish exhibiting symptoms of mycobacterial infection, signs of pain (as per humane end point) or discomfort were euthanized and collected for genotyping analysis. Symptoms included skin lesions, upturned scales, changes in swimming or behaviour, gasping for breath or lack of responsiveness.

### *M. marinum* infections

*M. marinum* (ATCC 927 strain) was used for all infections. The bacterial preparation and injection procedure have been described previously (Parikka et al., 2012). Briefly, the bacteria were inoculated from BD Difco Middlebrook 7H10 plates (BD Biosciences, Franklin Lakes, NJ, USA) into 7H9 media (BD Biosciences) and grown at 29°C protected from light. After 72 h, the culture was passaged to optical density at 600 nm (OD_600_)=0.07 and allowed to reach the logarithmic growth phase. For larval infections, bacteria were pelleted and resuspended in a desired volume of 2% polyvinylpyrrolidone in PBS. Phenol Red was used as a dye to visualize the injection, and a 1 nl injection volume was calibrated using a halocarbon oil droplet on a microscope scale bar. For survival analysis, larval zebrafish were injected with *M. marinum* or a PBS mock injection mix into the yolk sack at the two- to eight-cell stages, and dechorionated at 1 dpf. For bacterial burden assay, embryos were injected with *M. marinum* either at the two- to 1000-cell stage into the yolk sack or at 2 dpf into blood circulation valley. For adult fish, bacteria were pelleted and resuspended in PBS with 10% Phenol Red. Anaesthetized adult fish were injected with 5 µl of injection solution intraperitoneally using a 30 G needle. For both larval and adult zebrafish, injection doses were plated onto 7H10 plates (BD Biosciences) during the procedure to verify the dose.

### RNA extractions

Gene expression analysis of adult zebrafish tissues and larval zebrafish was done with qPCR. For organ collection, adult AB zebrafish were euthanized, and their organs were collected with tweezers into PBS-containing microcentrifuge tubes. Larval zebrafish were homogenized by pipetting. Zebrafish organs and larval zebrafish were homogenized by pipetting or with a needle and syringe. For RNA-seq analysis of kidney marrow and qPCR, kidneys were homogenized in tubes containing an RNA-preserving buffer and six ceramic beads (diameter of 2.8 mm, Omni International, Kennesaw, GA, USA) at 6.5 m/s for 2×30 s on dry ice with a FastPrep-24™ 5G bead-beating grinder and lysis system (MP Biomedicals, Irvine, CA, USA). For RNA-seq analysis of neutrophils, kidney marrow was homogenized by pipetting prior to cell sorting, and cell suspensions were homogenized using a needle and syringe. RNA extractions were done with RNeasy Mini Plus or Micro Plus kits (Qiagen, Hilden, Germany). For the RNA expression experiments in the organs of the adult zebrafish, DNA removal columns were not used; instead, the contaminating genomic DNA was removed with a Rapid Out DNA removal kit (Thermo Fisher Scientific).

### qPCR analyses

For qPCR, cDNA was synthesized with a Sensifast cDNA synthesis kit (Bioline, Meridian Bioscience, Newtown, OH, USA). Transcript quantitation was performed with PowerUp Sybr Green Master Mix (Thermo Fisher Scientific) with a CFX96 Real-time PCR detection system (Bio-Rad, Hercules, CA, USA). Primers for qPCR are provided in Table S5. Cycle threshold (Ct) values were normalized to the Ct values of the *eef1a1/1* transcript (Tang et al., 2007), and amplicon sizes were confirmed with a gel run. The program cycling parameters were 50°C 02:00 (min:s), 95°C 02:00, 40 cycles of 95°C 00:03 and 59-60°C 00:30, followed by a melt curve from 55°C to 95°C at 0.5°C increment. All samples were measured in duplicate.

### Determination of the bacterial burden

At 4 wpi, fish were euthanized with an overdose of Tricaine. Fish were pinned on a polystyrene piece, and the whole-organ block was released with tweezers and a spatula. The organ block was collected into a screw cap tube with six ceramic beads. Samples were homogenized in tubes containing the Tri-reagent (Molecular Research Center, Cincinnati, OH, USA) at 6.5 m/s for 2×30 s on dry ice with a FastPrep-24™ 5G bead-beating grinder and lysis system. DNA was extracted as in Parikka et al. (2012): *M. marinum* genome copies were quantitated with genome-specific primers (F, 5′-CACCACGAGAAACACTCCAA-3′; R, 5′-ACATCCCGAAACCAACAGAG-3′) against a standard curve (1:5 dilution series) of a sample of known bacterial burden. Measurement was done with a Sensifast Sybr no-ROX kit (Bioline) in a Bio-Rad CFX96 Real-time PCR detection system, with up to 1 µg DNA as template. The program used was 95°C 05:00, followed by 40 repeated cycles of 95°C 00:05, 65°C 00:10, 72°C 00:10, followed by a melting curve from 55°C to 95°C at 0.5°C increment. The correct size of the amplification product was confirmed with agarose gel electrophoresis. To determine the bacterial burden in larval zebrafish, larvae were euthanized at 4 or 5 dpi. After extracting DNA and genotyping larvae according to a protocol described for CRISPR-Cas9 mutagenesis and genotyping, the number of *M. marinum* copies was quantified as described above.

### Flow cytometry and cell-sorting experiments

Adult zebrafish were euthanized with an overdose of Tricaine anaesthetic. The fish was placed on a piece of polystyrene, cut with a scalpel and pinned open. To determine the blood cell population, the kidney was peeled from the cavity with tweezers and suspended in 100 µl PBS with 0.5% foetal bovine serum (FBS) that was kept on ice. The suspended kidney was then homogenized by pipetting, vortexed briefly and passed through a 35 µm cell strainer using a syringe plunger to help push the cells through into a microcentrifuge tube. The strainer was rinsed with extra buffer. Samples were kept on ice.

The viability stain FVS 510 (BD Biosciences) was used for excluding dead cells from the analysis. Briefly, cells were suspended in 2 ml PBS, pelleted at 400 ***g*** for 5 min. The supernatant was removed, and the pellet was resuspended in 1 ml PBS. Then, 1 µl of stain was added, and tubes were gently vortexed. Samples were incubated for 30 min. Cells were washed twice with 2 ml PBS with 0.5% FBS. Finally, cells were resuspended in PBS with 0.5% FBS. Just before sorting, samples were passed through a strainer cap of a FACS tube by centrifugation to ensure that there were no clumps (400 ***g***, 00:30). Flow cytometry and sorting were done with a FACS Arya Fusion (BD Biosciences). Cells were gated according to Langenau et al. (2004).

For each sample, 20,000 events were recorded. Sorted cells were kept on ice and pelleted after the flow cytometry was finished. RNA was extracted with a RNeasy Micro Plus Kit (Qiagen). Flow cytometry results were analysed with FlowJo 10.7.1.

To sort out neutrophils for RNA-seq, fish homozygous for *pycard^tpu4^* or *pycard^tpu5^* and WT controls crossed to *Tg(mpx:GFP)i114* fish with fluorescent neutrophils were euthanized and dissected as described above. Dissected kidneys were suspended in 200 µl PBS with 1% FBS and 25 µM HEPES and kept on ice. Suspended kidneys were homogenized by pipetting and vortexing briefly, before passing them through a 35 µm cell strainer by spinning down. To exclude dead cells from the analysis, 5 µl Propidium Iodide Staining Solution (Thermo Fisher Scientific) was added, and samples were incubated for 5 min.

Flow cytometry and cell sorting were done with a FACS Arya Fusion (BD Biosciences), based on fluorescence (GFP) emitted by neutrophils. Cells were gated according to Fig. S9A. All events for each sample were recorded, and sorted neutrophils were suspended in PBS with 1.5% FBS and 25 µM HEPES. Cell suspensions were kept on ice, and RNA was extracted with a RNeasy Micro Plus Kit (Qiagen).

### Histology

Eight-month-old fish were injected with a low dose of *M. marinum* and euthanized at 8 wpi with an overdose of Tricaine. Fish were fixed in 10% phosphate-buffered formalin at room temperature in a rotator for 7 days after the removal of the heads and tails. Decalcification was carried out by incubating the samples for 7 days at room temperature in 0.5 M ethylenediaminetetraacetic acid (pH 8.0) in dH_2_O. Samples were incubated in 70% ethanol overnight at room temperature with stirring, after which they were carried through a rising ethanol series and transferred to xylene. Samples were then cast in paraffin and cut with a microtome (SM2010R, Leica, Wetzlar, Germany), starting from the dorsal side. Four 5 µm histological sections were collected at the intervals of 200 µm on StarFrost advanced adhesive (76×26 mm) glasses (Waldemar Knittel Glasbearbeitungs GmbH, Braunschweig, Germany). Samples were deparaffinized and used for either Ziehl–Neelsen, trichrome or hypoxia staining. Ziehl–Neelsen staining was carried out according to a standard protocol, and trichrome staining was performed as described in Myllymäki et al. (2018). After staining with Stainmate (Thermo Fisher Scientific), the slides were dehydrated with a series of ethanol solutions of increasing concentrations ending with xylene and embedded with DPX new (Sigma-Aldrich, St Louis, MO, USA). All slides were scanned with a NanoZoomer S60 digital slide scanner (Hamamatsu, Hamamatsu City, Japan) and analysed with NDP View (Hamamatsu). Granulomas were counted and analysed based on their diameter, location, structure and hypoxicity.

Tissue hypoxia was detected with a Hypoxyprobe-1 kit (HP1-100Kit, Hypoxyprobe, Burlington, MA, USA). Pimonidazole hydrochloride (part of the HP1-100 kit) dissolved in PBS was injected intraperitoneally during terminal anaesthesia. Glasses were pre-treated with 0.05 M Tris – 0.01 M EDTA buffer with 0.05% Tween 20 (pH 9) using Lab Vision PT Module (Thermo Fisher Scientific). Endogenous peroxidase activity was blocked with a 5 min incubation in 3% hydrogen peroxidase (23614.291, VWR, West Chester, PA, USA) and further by treating the samples with Bloxall blocking solution (SP-6000, Vector Laboratories, Burlingame, CA, USA) for 10 min. Samples were incubated with a 1:600 dilution of Hypoxyprobe-1 Mab1 (part of the HP1-100 kit) for 30 min, after which they were treated with a secondary antibody, universal immune-peroxidase polymer anti-mouse complex (414131F, Nichirei Biosciences, Tokyo, Japan) for 30 min. Staining was carried out with a 10 min incubation with Histofine DAB-2V (425314F, Nichirei Biosciences) and counterstaining with a 2 min incubation with Mayers Hematoxylin Plus (01825, Histolab Products AB, Askim, Sweden). An Autostainer 480 (Lab Vision, Thermo Fisher Scientific) was used to perform the staining. The glasses were dehydrated, embedded and scanned as described above.

To analyse cell death and to detect apoptotic cells in granulomas, TUNEL assay was performed with a Click-iT TUNEL Colorimetric IHC Detection Kit (Thermo Fisher Scientific). Histological samples prepared as described previously were deparaffinized and stained according to the manufacturer's protocol. Counterstaining with 1 min incubation was performed manually with 1:5 diluted Mayers Hematoxylin Plus (Histolab Products AB), and dehydrating, embedding and scanning of the samples was carried out as described previously. The number of granulomas with apoptotic cells was manually calculated with NDP View.2 (Hamamatsu).

### RNA-seq

For RNA-seq of kidney (the main hematopoietic organ in zebrafish), zebrafish were infected with a low dose of *M. marinum* or mock injected with PBS. At 4 wpi, fish were euthanized and kidneys were collected as described for the flow cytometry and cell-sorting experiments. Kidneys were homogenized with ceramic beads as explained before, in RNA-preserving buffer RLT plus with β-mercaptoethanol (Sigma-Aldrich). RNA was extracted with a RNeasy Plus Mini kit (Qiagen). RNA quality was assessed with a Fragment analyzer (Agilent, Santa Clara, CA, USA). RNA-seq services were provided by the Finnish Centre for Functional Genomics, Turku, Finland. The run was performed with NovaSeq 6000 S4 v1.5. (paired-end sequencing, 100 bp read length, 20 million reads per sample depth). RNA-seq data are available at NCBI's Gene Expression Omnibus (GEO) (Barrett et al., 2013; Edgar et al., 2002) under the identifier GSE189627.

Quality control for the RNA-seq read data was done using FastQC version 0.11.7. The reads were aligned using STAR aligner (Dobin et al., 2013) version 2.5.3a and the Ensembl reference genome GRCz11. Read counts for genes were quantified using featureCounts (Liao et al., 2014) version 1.6.2 and the Ensembl reference gene set release GRCz11.104 (Hubbard et al., 2002). A differential expression analysis was conducted using R version 3.6.1 and DESeq2 (Love et al., 2014) version 1.24.0. Differentially expressed genes were determined as genes that have a *P*-value <0.05 after adjustment for multiple testing, |log2 fold change| >1 and an absolute median difference of library size-normalized read counts >13 between two conditions. Gene expression heat maps were generated with pheatmap version 1.0.12. Differentially expressed genes were classified based on the data available from the Ensembl genome browser (Howe et al., 2021) version 104, Reactome (Griss et al., 2020), ZFIN (Ruzicka et al., 2019) and the literature.

For RNA-seq analysis of neutrophils, *pycard^tpu4/tpu4^*, *pycard^tpu5/tpu5^* and WT controls [*pycard^tpu4^* and *pycard^tpu5^* originally crossed to transgenic *Tg(mpx:GFP)i114 (AB)* fish] were infected with *M. marinum* [mean dose, 95 CFU; range, 69-118 CFU (*pycard^tpu4^*); mean dose 6 CFU; range, 4-8 CFU (*pycard^tpu5^*)]. At 4 wpi, fish were euthanized, and kidney marrow-derived neutrophils were obtained as described for flow cytometry and cell-sorting experiments. RNA from neutrophils was extracted with a RNeasy Micro Plus Kit (Qiagen), and quality of RNA was assessed with a NanoDrop spectrophotometer (Thermo Fisher Scientific).

RNA-seq service and bioinformatic analysis were performed by Novogene, Cambridge, UK. Library construction was performed on the Illumina platform (paired-end sequencing, 150 bp read length) yielding >20 million reads per sample. The reads were aligned using HISAT2 (Mortazavi et al., 2008) and Ensembl reference genome GRz11, gene set releases grcz11_gca_000002035_4 (Hubbard et al., 2002) (*pycard^tpu4^*) and ensembl_109_danio_rerio_grcz11_primary (Hubbard et al., 2002) (*pycard^tpu5^*). Analysis of differentially expressed genes was performed with DESeq2 (Anders and Huber, 2010). RNA-seq datasets of *pycard^tpu4^* and *pycard^tpu5^* are available at GEO (Barrett et al., 2013; Edgar et al., 2002) under the identifiers GSE270136 and GSE287594, respectively.

For further analysis, genes with at least two samples with ≥20 normalized reads, |log2 fold change| >1 and |log2 fold change of medians| >1 between the groups were included. Genes were classified based on the data available from the Ensembl genome browser (Martin et al., 2023) versions 111, 112 and 113, ZFIN (Ruzicka et al., 2019), GeneCards (Stelzer et al., 2016) and the literature (Table S4).

### Imaging and quantifying myeloid cells

To detect the effect of the loss of *pycard* post wounding, offspring of heterozygous *pycard^tpu4^* mutants carrying fluorescent neutrophils [homozygous mutants crossed to *Tg(mpx:GFP)*] or macrophages [homozygous mutants crossed to *Tg(mpeg:GFP)*] was dechorionated and transferred to embryonic medium supplemented with 0.0045% N-phenylthiourea (PTU; P7629, Sigma-Aldrich) at 1 dpf. At 2 dpf, fluorescent larvae were picked using an AZ100 macroscope (Nikon, Tokyo, Japan). At 3 dpf, the larvae were wounded under a Stemi DV4 microscope (Carl Zeiss AG, Oberkochen, Germany) with a Fine-Ject 30 G/0.3×12 mm needle (4710003012, Henke Sass Wolf GmbH, Tuttlingen, Germany) on the side of the tail fin, embedded in 1.7% or 2% methyl cellulose (M0512-100G, Sigma-Aldrich) and imaged in one representative focus layer with the Nikon AZ100 macroscope and NIS-Elements D 5.02.00 software (Nikon) 1.5-3 h post wounding. The exposure time on the green channel was set to 125 ms for *Tg(mpx:GFP)* and to 600 ms or 1000 ms for *Tg(mpeg1.1:GFP)*. The figures were saved as tiff files. Fluorescent neutrophils and macrophages were manually calculated from unmodified figures at 120% or 150% magnification, respectively, using Corel PaintShop Pro 2020 version 22.0.0.132 (Alludo, Ottawa, Canada). The larvae were genotyped as described in the ‘CRISPR-Cas9 mutagenesis and genotyping’ section.

To analyse the effect of *pycard* deficiency on neutrophil and macrophage count during *M. marinum* infection, embryos originating from heterozygous *pycard^tpu4^* and *pycard^tpu5^* parents carrying fluorescent neutrophils or macrophages were dechorionated at 1 dpf and infected with *M. marinum* into blood circulation valley at 2 dpf. After infecting, embryos were transferred to embryonic medium with 0.0045% PTU and imaged at 1 and 2 dpi with the Nikon AZ100 macroscope, using NIS-Elements D software 5.02.00. Exposure time on the green channel was set to 1000 ms, and images were saved as nd2 and exported as tiff with NIS-Elements Viewer 5.22.00 (Nikon). Neutrophils and macrophages were manually calculated from unmodified figures at 100% or 120% magnification, respectively, using Corel PaintShop Pro 2020 version 22.0.0.132, and larvae were genotyped as described previously.

### Power calculations and statistical analysis

Based on our previous data, we estimated the number of animals required for the survival and bacterial burden experiments (Harjula et al., 2018, 2020; Myllymäki et al., 2018; Ojanen et al., 2015; Parikka et al., 2012). With the ClinCalc Sample Size Calculator, the required group size was estimated to be 16-20 fish (alpha, 0.05; power, 0.8). As the experiments were performed unaware of genotype, a moderately higher group size of 25 was selected so that the group contained a sufficient number of each genotype. Log-rank (Mantel–Cox) test was used to determine statistical significance for the survival experiments. Statistical analyses were performed with Graph Pad Prism 5.02. *P*<0.05 was considered significant.

## Supplementary Material

10.1242/dmm.052061_sup1 Supplementary information
