# Supplementary material for: The inflammasome adaptor pycard is essential for immunity against Mycobacterium marinum infection in adult zebrafish
Source: Dis Model Mech. 2025 Mar 24;18(9):dmm052061. doi: 10.1242/dmm.052061 (PMC11972081; doi:10.1242/dmm.052061)
Supplement: Supplementary information [file dmm-18-052061-s1.pdf]

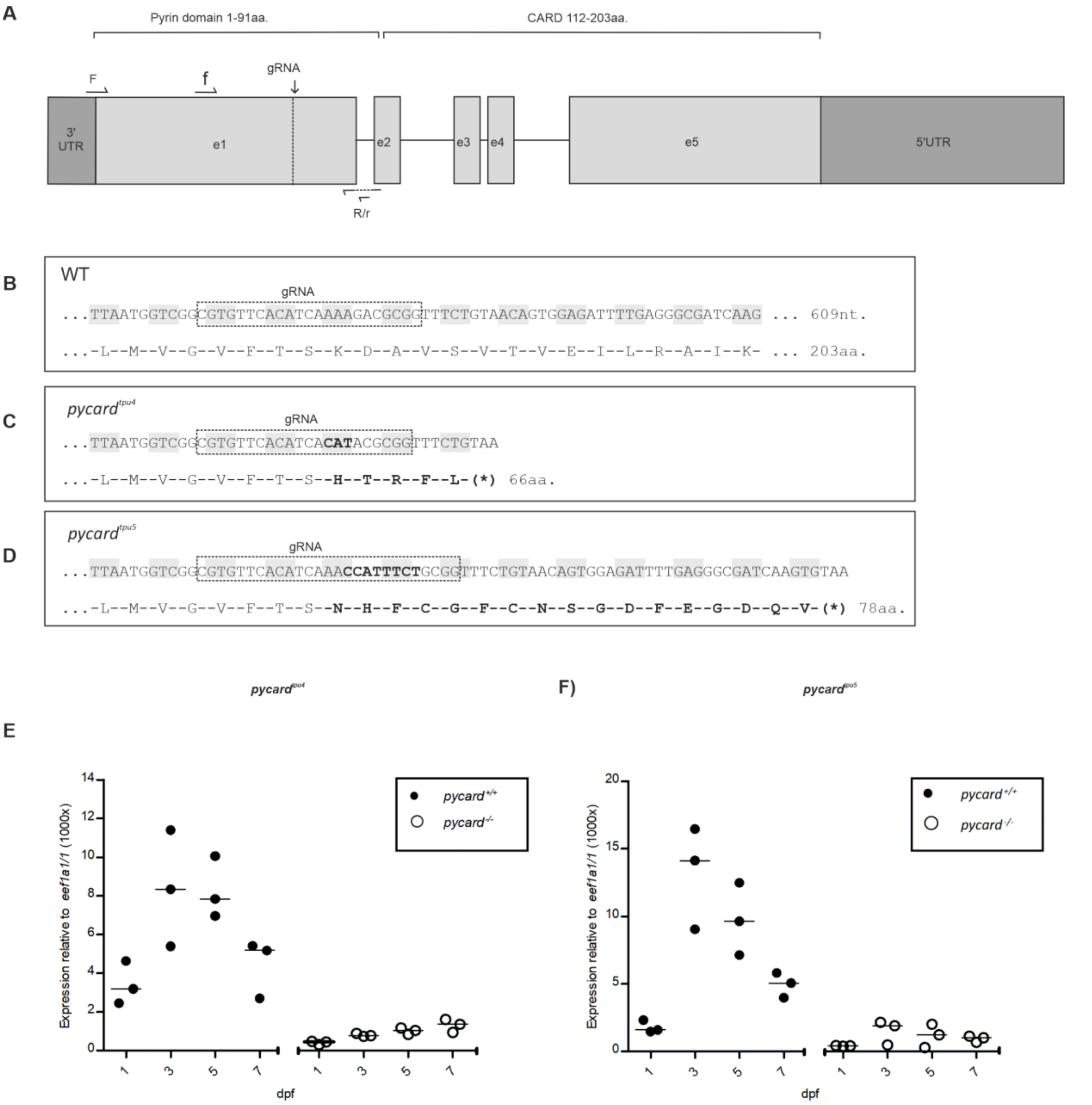

Fig. S1. Generation of *pycard* mutants with CRISPR-Cas9.

A) The structure of the zebrafish *pycard* gene is presented with a box indicating the gRNA binding site. Exons 1-5 are indicated with e1-e5, introns (not drawn to scale) have been indicated with horizontal lines joining the exons, and the untranslated regions (3'UTR and 5'UTR) are indicated with a darker grey colour. The primers used in genotyping are indicated with a half arrow and a capital letter (F=forward primer, R=reverse primer), primers for transcript quantitation are indicated with lower case letter (f=forward primer, r=reverse primer). The reverse primer for quantitation is designed across the intron, as indicated by a dashed line over the intron. Below, the B) WT and C) the mutant *pycard*^{tpu4} (left) and D) the mutant *pycard*^{tpu5} DNA and coding sequences are shown underlined, with a box indicating the gRNA binding site and amino acids resulting from the frame shift are bolded. The length of the resulting amino acid chain is marked at the end. E-F) The gene expression was measured with qPCR in *pycard*$^{+/+}$ and *pycard*$^{-/-}$ larvae at 1, 3, 5 and 7 days postfertilization (dpf) for E) the mutant line *pycard*^{tpu4} F) mutant line *pycard*^{tpu5}. Results were normalized to *eef1α* expression. Each sample consists of 3-5 pooled larvae (n=3/timepoint). The line indicates median.

Disease Models & Mechanisms • Supplementary information

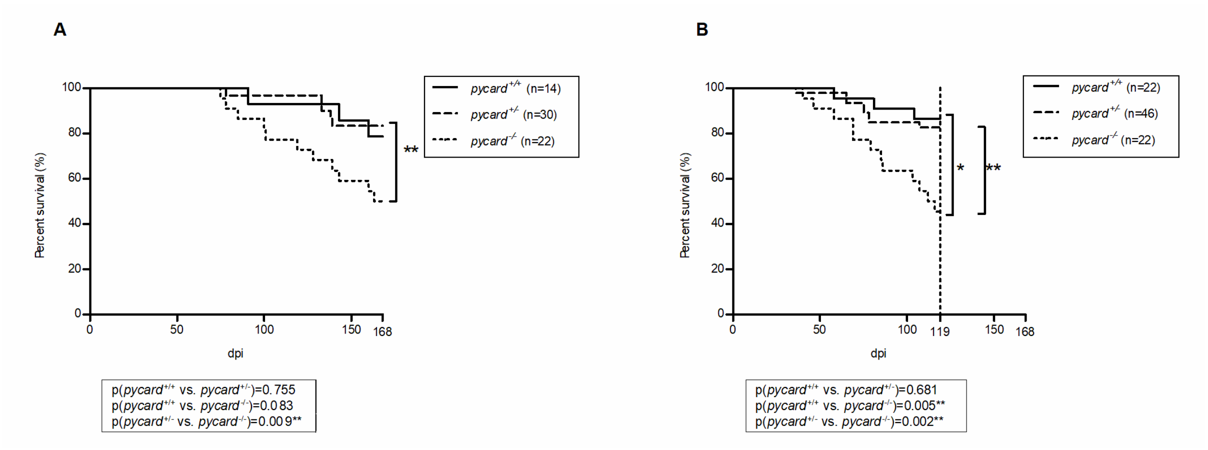

Fig. S2. The *pycard^{tpu5}* mutants are susceptible for *M. marinum* infection.

Adult fish of the *pycard^{tpu5}* line were infected with a low dose of *M. marinum* and their survival was followed daily. A) The first experiment, mean dose 14 CFU, range 9-18 CFU. End point survival proportions *pycard$^{+/+}$*: 78.6%, *pycard$^{+/tpu5}$*: 83.3%, *pycard$^{tpu5/tpu5}$*: 50.0%. B) Mean dose 40 CFU, range 25-57 CFU. End point survival proportions *pycard$^{+/+}$*: 86.4%, *pycard$^{+/tpu5}$*: 82.6%, *pycard$^{tpu5/tpu5}$*: 45.5%. The fish were genotyped post-mortem. The survival data are presented as a Kaplan-Meier survival curve. The statistical analysis was done with a log rank test. The second survival experiment b) was terminated earlier to minimize unnecessary suffering.

Disease Models & Mechanisms • Supplementary information

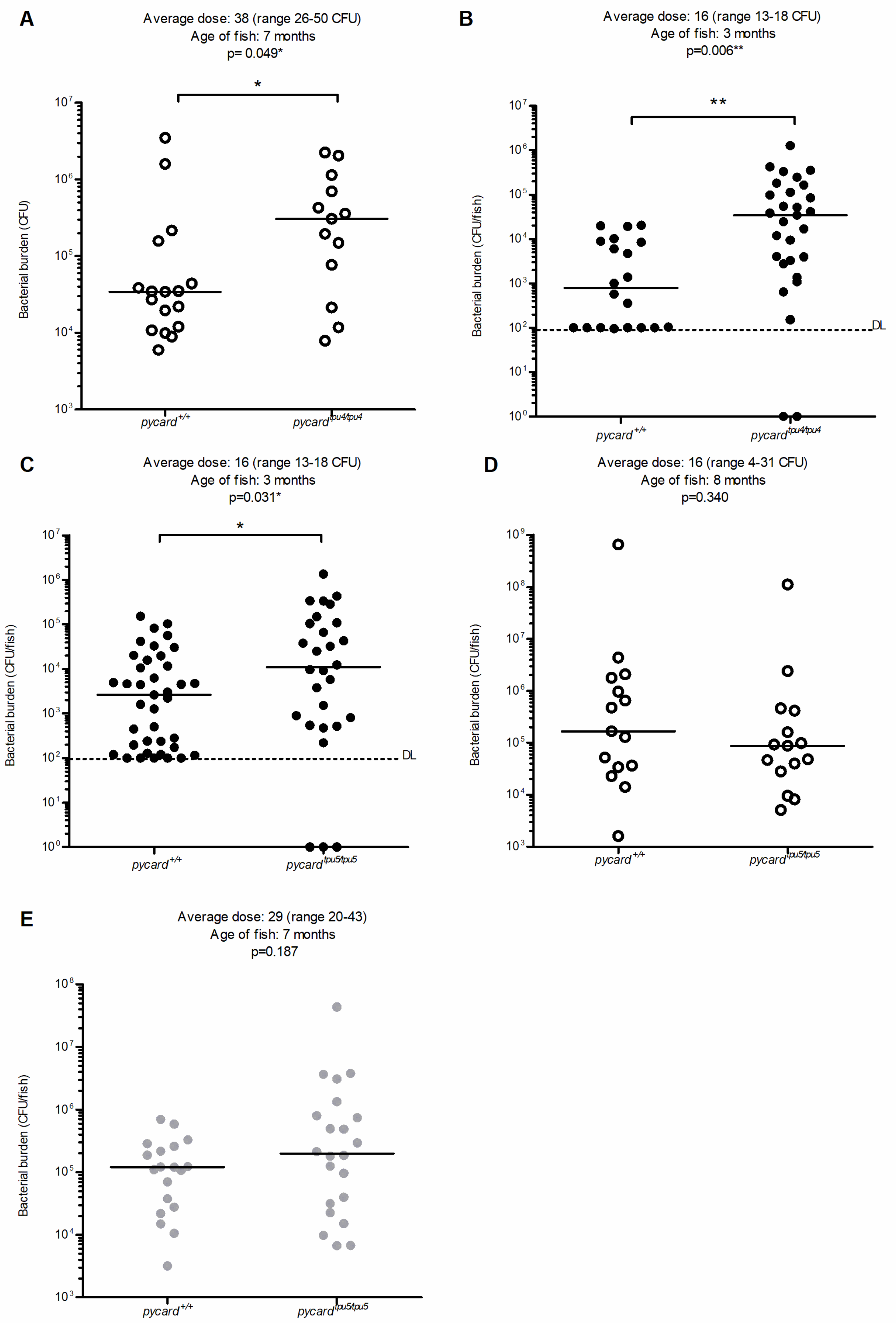
A
Average dose: 38 (range 26-50 CFU)
Age of fish: 7 months
p= 0.049*
*
Bacterial burden (CFU)
pycard+/+
pycardtpu4/tpu4
B
Average dose: 16 (range 13-18 CFU)
Age of fish: 3 months
p=0.006**
**
Bacterial burden (CFU/fish)
DL
pycard+/+
pycardtpu4/tpu4
C
Average dose: 16 (range 13-18 CFU)
Age of fish: 3 months
p=0.031*
*
Bacterial burden (CFU/fish)
DL
pycard+/+
pycardtpu5/tpu5
D
Average dose: 16 (range 4-31 CFU)
Age of fish: 8 months
p=0.340
Bacterial burden (CFU/fish)
pycard+/+
pycardtpu5/tpu5
E
Average dose: 29 (range 20-43)
Age of fish: 7 months
p=0.187
Bacterial burden (CFU/fish)
pycard+/+
pycardtpu5/tpu5

Fig. S3. ***pycard***$^{-/-}$ **adult fish display a higher bacterial burden.**

Zebrafish from both mutant lines were infected with a low dose of *M. marinum*, and the bacterial burden was analysed at 4 wpi in whole organ block DNA using qPCR with *M. marinum* genome specific primers. A) *pycard*^{tpu4}, medians for both groups *pycard*$^{+/+}$: 34129 CFU, *pycar*$^{tpu4/tpu4}$: 306007 CFU, p=0.049* (n(WT)=17, (*pycard*$^{tpu4/tpu4}$)=13). B) *pycard*^{tpu4}, medians for both groups *pycard*$^{+/+}$: 792 CFU, *pycard*$^{tpu4/tpu4}$: 34612 CFU, p=0.006** (n(WT)=20, (*pycard*$^{tpu4/tpu4}$)=29). C) *pycard*^{tpu5}, medians for both groups *pycard*$^{+/+}$: 2636 CFU, *pycard*$^{tpu5/tpu5}$: 10992 CFU, p=0.031* (n(WT)=39, (*pycard*$^{tpu5/tpu5}$)=28), D) *pycard*^{tpu5}, *pycard*$^{+/+}$: 167027 CFU, *pycard*$^{tpu5/tpu5}$: 87447 CFU, p=0.340 (n(WT)=15, (*pycard*$^{tpu5/tpu5}$)=15), E) *pycard*^{tpu5}, *pycard*$^{+/+}$: 120490 CFU, *pycard*$^{tpu5/tpu5}$: 199455 CFU, p=0.187 (n(WT)=19, (*pycard*$^{tpu5/tpu5}$)=22). The line indicates the median, the data were analysed with the Mann-Whitney U-test, two tailed. For statistical reasons, samples whose quantitation failed or was below 100 CFU detection limit (DL), a value of 100 CFU for *pycard*$^{+/+}$ and 0 CFU for *pycard*$^{-/-}$ were given.

Disease Models & Mechanisms • Supplementary information

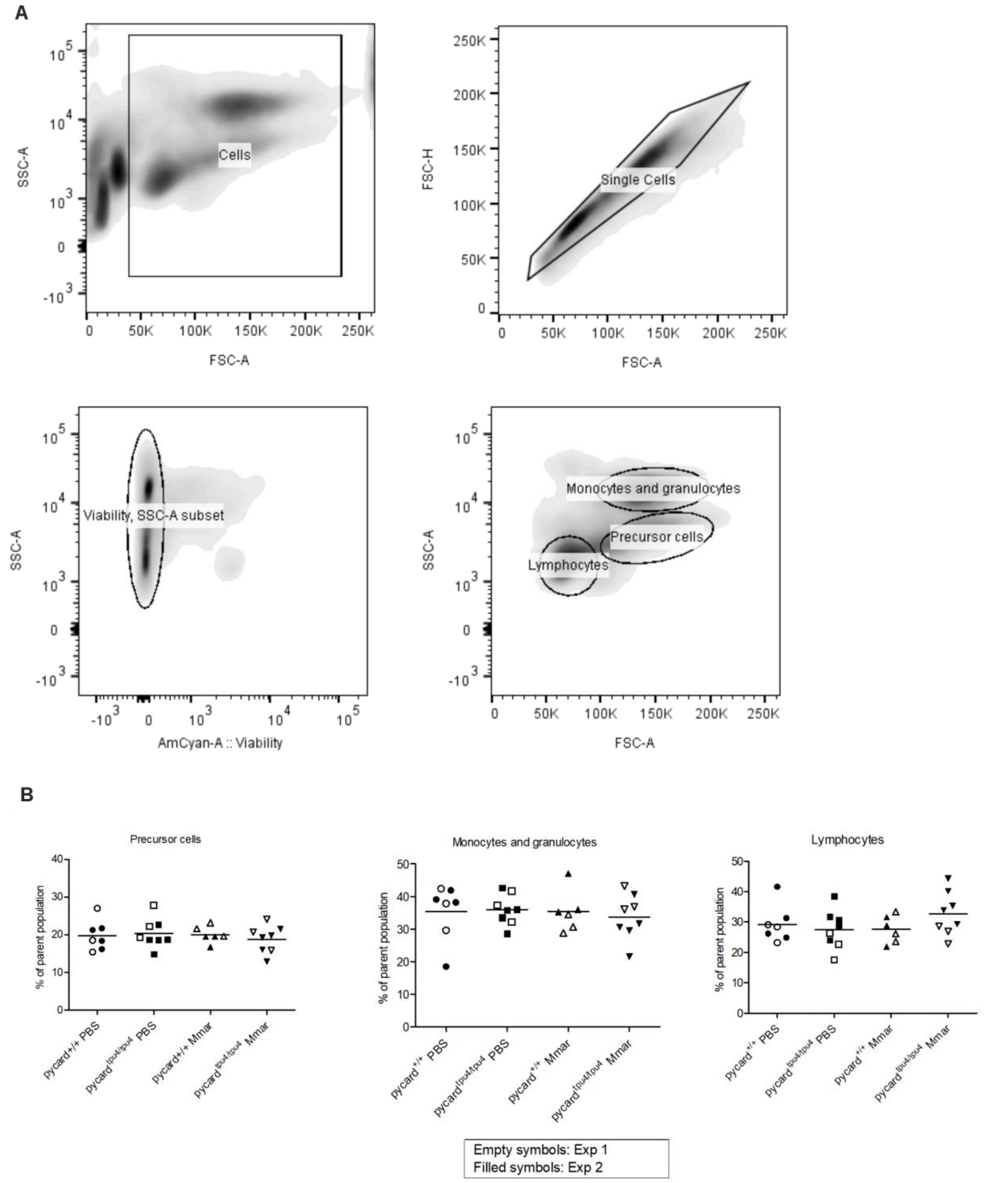

Fig. S4. Flow cytometry gating strategy and results from individual experiments.

A) Cells were gated from debris using forward scatter (FSC-A) vs. side scatter (SSC-A). Single cells were gated from all cells using FSC-A vs. FCS-H (pulse geometry gating). Live cells were gated based on the FVS510 live-dead stain. Leukocyte populations were gated based on size (FSC-A) and granularity (SSC-A). B) The experiment was repeated twice, and individual experiments are shown with either empty or filled symbols (n=3+4(*pycard*$^{+/+}$ PBS), n=3+5(*pycard*$^{tpu4/tpu4}$ PBS), n=3+4(*pycard*$^{+/+}$ *M. marinum*), n=3+5(*pycard*$^{tpu4/tpu4}$ *M. marinum*). 20 000 events were recorded per sample. Bacterial burden in experiment 1, mean dose 17 CFU, range 12-28 CFU. Bacterial burden in experiment 2, mean 19 CFU, range 16-21 CFU.

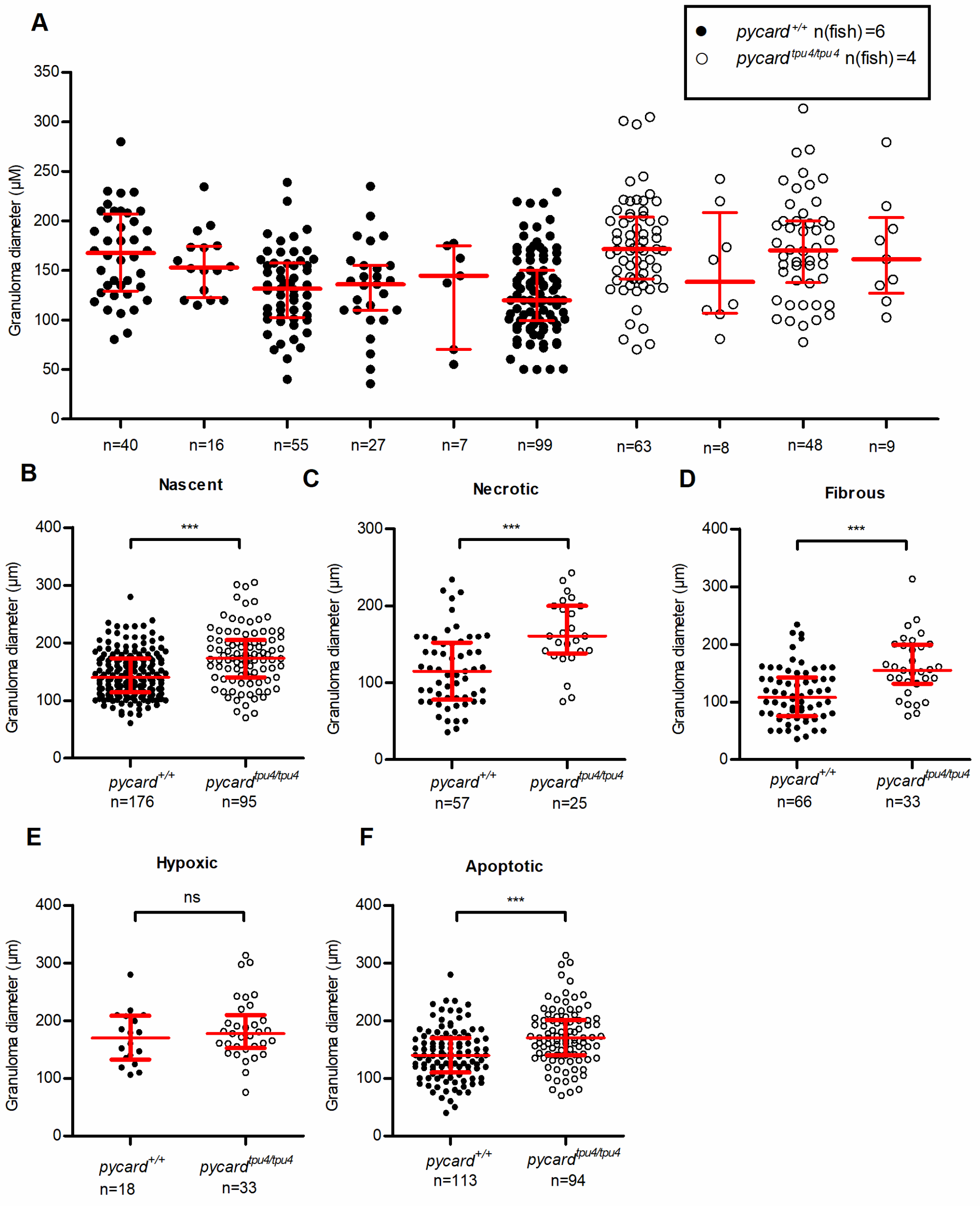

Fig. S5. Granuloma sizes per individual fish.

A) Zebrafish of the mutant line *pycard*^{tpu4} were infected with a low dose of *M. marinum* and at 8 wpi were sacrificed for analysis. All granulomas from each fish were analysed for their size. A linear mixed model was used for determining the effect of genotype on granuloma size (R-package lme4, fish as a random and genotype as a fixed factor). B-F) Granuloma size was analysed for each granuloma type separately. The data were analysed with Mann-Whitney U-test, two tailed. The lines indicate median and the interquartile range.

Disease Models & Mechanisms • Supplementary information

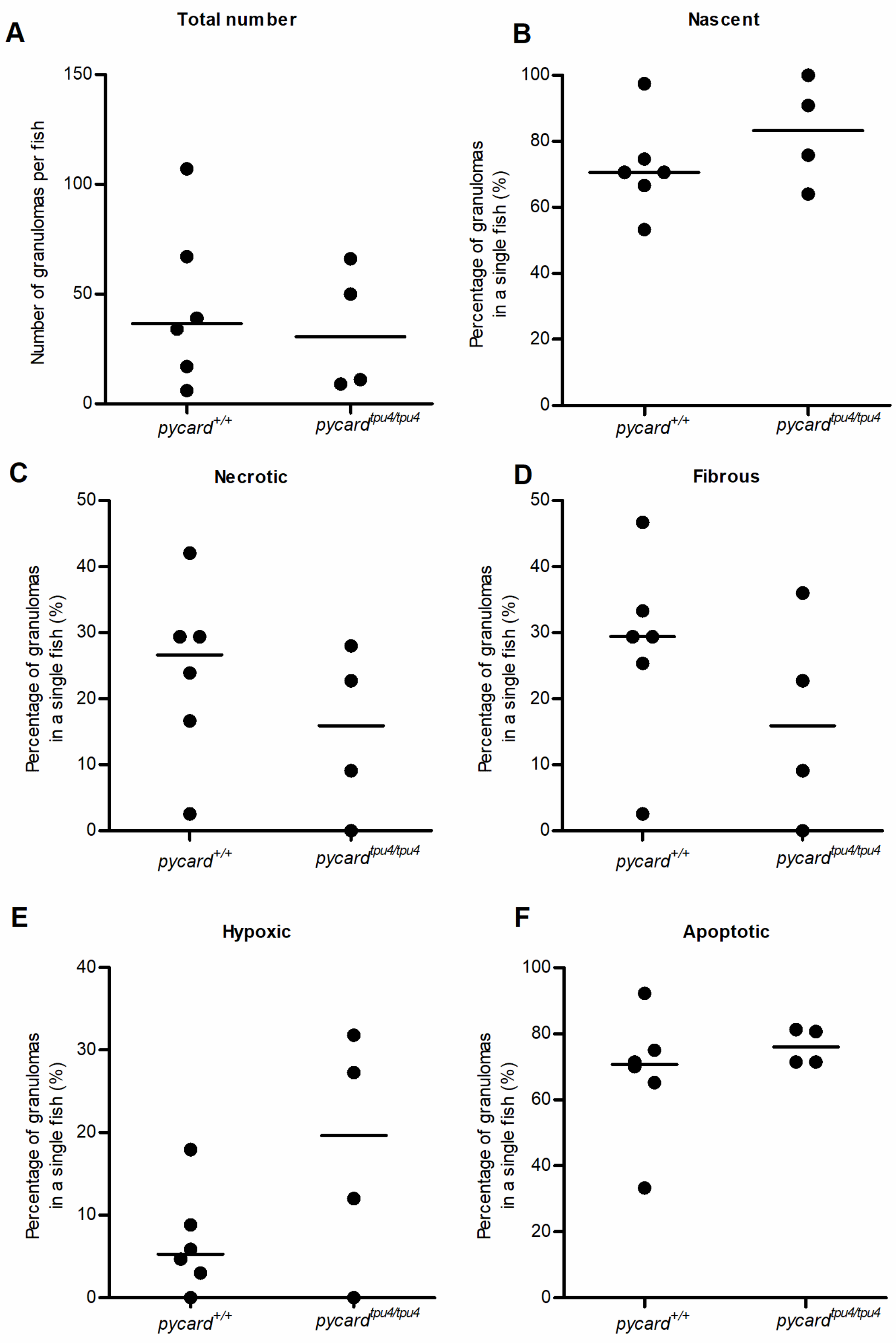

Fig. S6. Results from the granuloma characterization.

Each granuloma from the fish from the *pycard*^{tpu4} line (n=4(*pycard*$^{tpu4/tpu4}$), n=6(*pycard*$^{+/+}$) was A) quantitated and characterised for whether it presented features of a B) nascent, C) necrotic, D) fibrous capsule, E) surrounding hypoxia, F) containing apoptotic cells. The fraction of each type from the total number of granulomas in a fish is presented for both genotypes. No significant differences were observed.

Disease Models & Mechanisms • Supplementary information

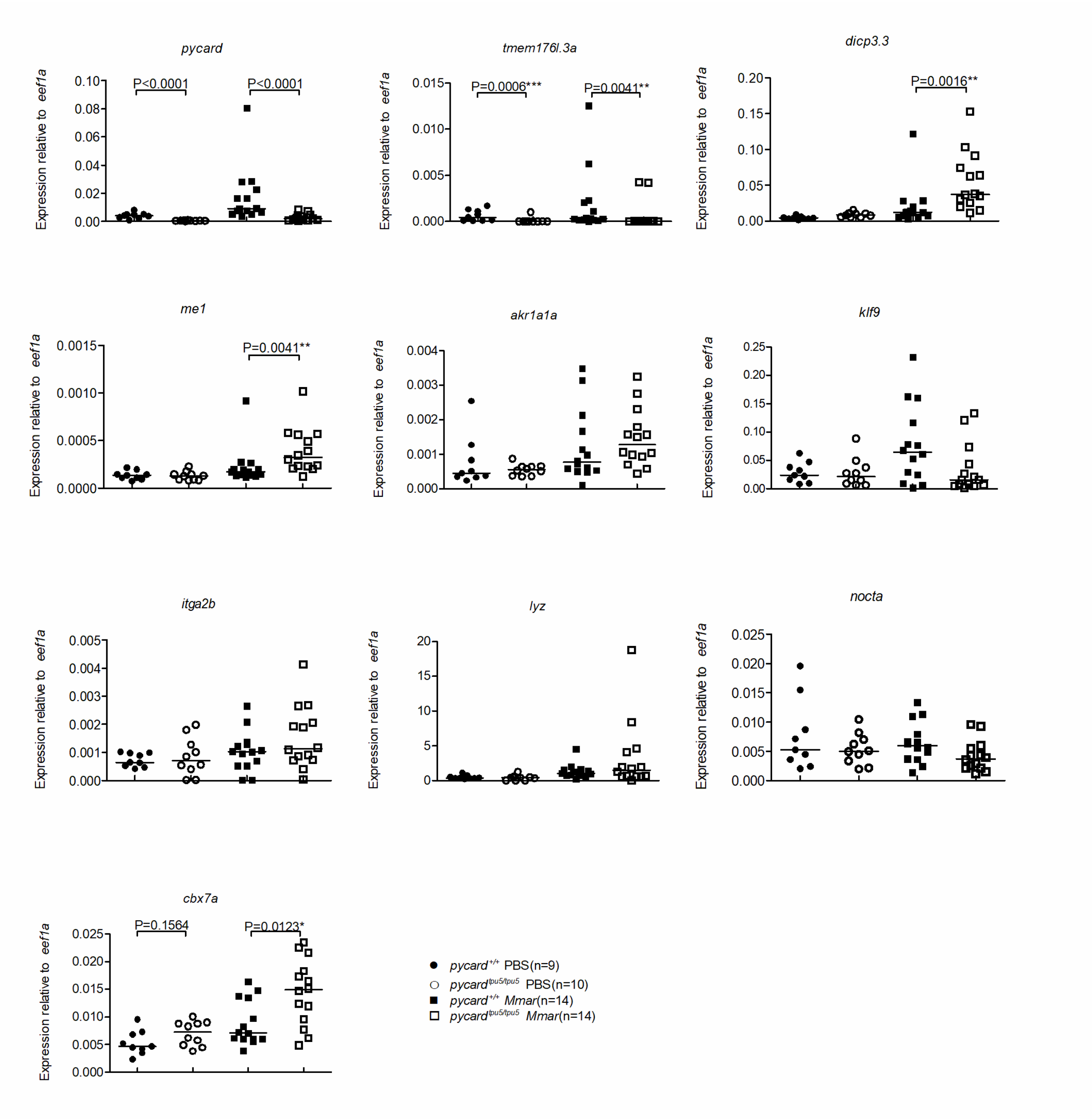

Fig. S7. Quantitative PCR analysis of selected genes in *pycard*^{tpu5} mutant line. Zebrafish were either mock injected or infected with a low dose of *M. marinum* (Mean dose 24 CFU, range 16-32 CFU). Both sexes were included in the experiment in approximately equal numbers. At 4 wpi, the fish were sacrificed and their kidney collected for RNA extraction. Transcripts were quantitated with qPCR. The line indicates the median.

Disease Models & Mechanisms • Supplementary information

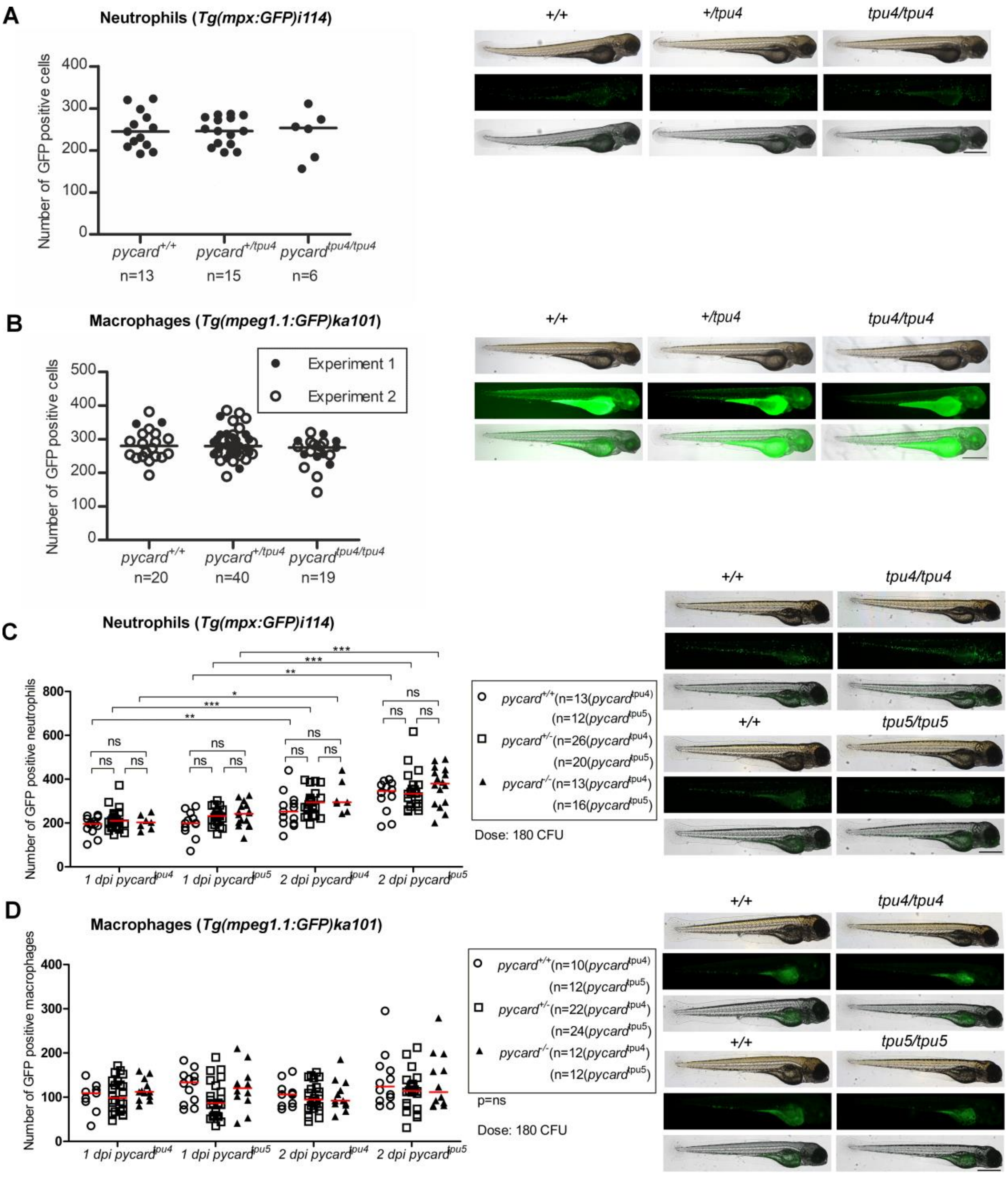

Disease Models & Mechanisms • Supplementary information

Fig. S8. Analysis of the number of neutrophils and macrophages in *pycard*^{tpu4} and *pycard*^{tpu5} mutants.

pycard$^{tpu4/tpu4}$ and *pycard*$^{tpu5/tpu5}$ zebrafish were crossed to transgenic *Tg(mpx:GFP)i114 (AB)* and *Tg(mpeg1.1:GFP)ka101 (AB)* zebrafish lines to receive *pycard*$^{+/tpu4}$ *and pycard*$^{+/tpu5}$ zebrafish with fluorescent macrophages or neutrophils, respectively. A-B) These fish were incrossed and at 3 dpi the progeny was wounded and and imaged with Nikon AZ100 macroscope and NIS-Elements D 5.02.00 software. The neutrophils and macrophages were manually counted from the figures using Corel PaintShop Pro 2020 version 22.0.0.132. The loss of *pycard* did not affect the number of A) neutrophils or B) macrophages in the larvae post wounding. The data in figure B) are pooled from two experiments. A two-tailed Mann-Whitney test was used for the statistical comparison of differences. In A-B) Representative images are shown from each group. Brightness and contrast were adjusted to the same level for the larvae with fluorescent neutrophils and for the larvae with fluorescent macrophages, the channels were merged and the figures flipped and/or rotated if needed with Fiji (Image J) (Schindelin et al., 2012). The scale bar is 500 µm. C-D) Progeny of incrossed fish were infected with *M. marinum* (average dose 180 CFU, range 97-330 CFU) at 2 dpf into blood circulation valley. At 1 dpi and 2 dpi, larvae were imaged and neutrophils and macrophages counted as described above. Within timepoints, there was no difference in the number of C) neutrophils or D) macrophages in *pycard* mutants when compared to WT larvae. C) Between timepoints, differences in the number of neutrophils were statistically significant within each genotype. Statistical significance of the results was analysed with Wilcoxon test. The line represents the median. In C-D) Representative images are shown from WT and *pycard*$^{-/-}$ groups. Brightness and contrast were adjusted to the same level for the larvae with fluorescent neutrophils and for the larvae with fluorescent macrophages, the channels were merged and figures flipped if needed with Fiji (Image J) (Schindelin et al., 2012) and/or rotated if needed with CorelDRAW 25.0.301 (Ottawa, Canada). The scale bar is 500 µm.

Disease Models & Mechanisms • Supplementary information

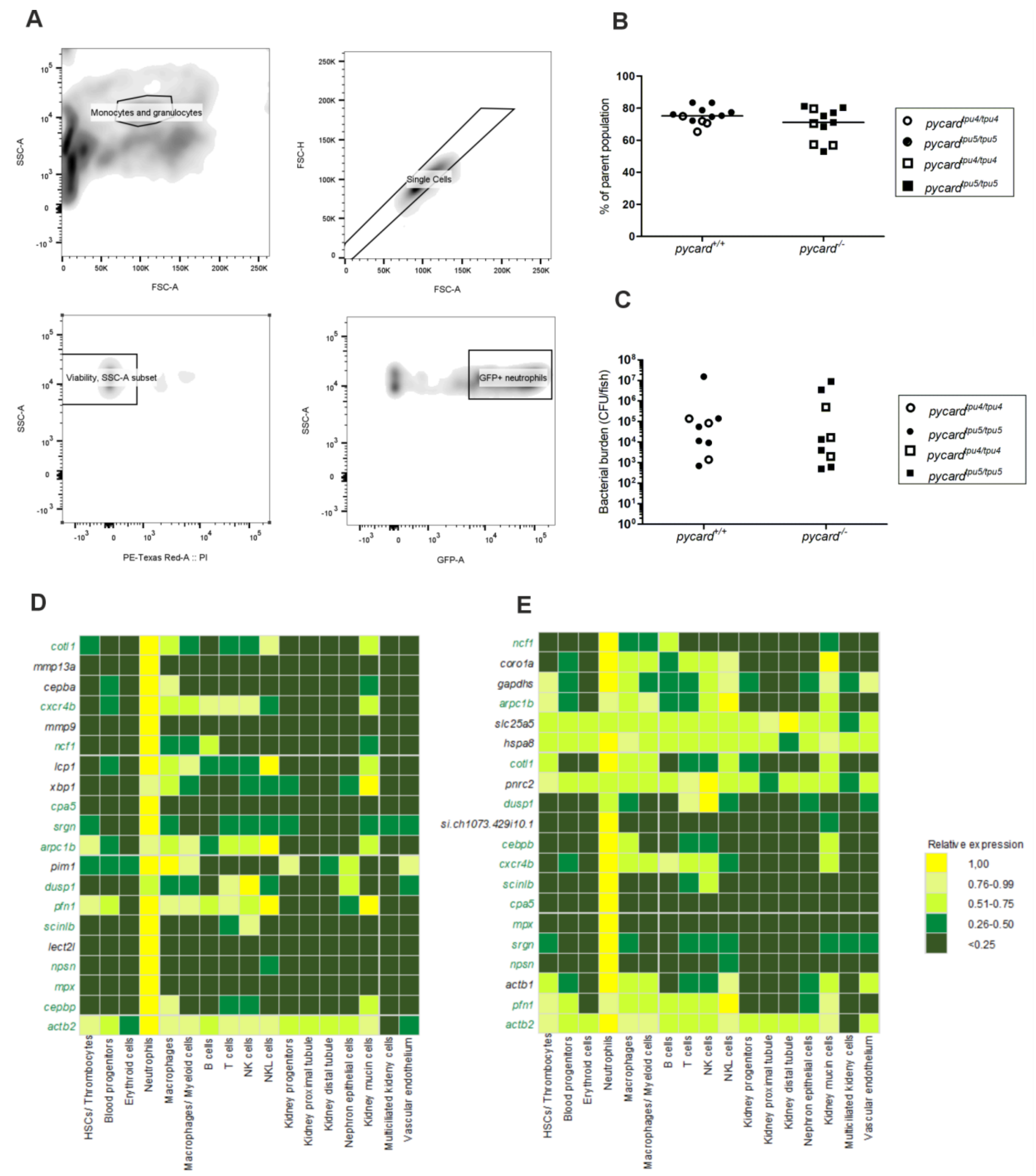

Disease Models & Mechanisms • Supplementary information

Fig. S9. Flow cytometry gating strategy, bacterial burden and 20 most expressed genes in WT fish derived neutrophils upon *M. marinum* infection (4 wpi).

A) Cells were gated from debris using forward scatter (FSC-A) vs. side scatter (SSC-A). Single cells were gated from all cells using FSC-A vs. FCS-H (pulse geometry gating). Live cells were gated based on the Propidium Iodide Staining Solution. Neutrophil populations were gated based on fluorescence (GFP), size (FSC-A) and granularity (SSC-A). 20 000 events were recorded per sample. Bacterial dose in the experiment, mean 95 CFU, range 69-118 CFU (*pycard*^{tpu4}); mean 6, range 4-8 CFU (*pycard*^{tpu5}). B) Percentage of neutrophils of total cell population. For *pycard*^{tpu4}, n=4(*pycard*$^{tpu4/tpu4}$, *pycard*$^{+/+}$); for *pycard,* ^{tpu5} n=7(*pycard*$^{tpu5/tpu5}$, n=8(*pycard*$^{+/+}$). The line represents the median. C) Bacterial burden in *M. marinum* infected *pycard*$^{+/+}$, *pycard*$^{tpu4/tpu4}$ and *pycard*$^{tpu5/tpu5}$ fish (*pycard*^{tpu4} and *pycard*^{tpu5} lines crossed to *Tg(mpx:GFP)i114* with fluorescent neutrophils). For *pycard*^{tpu4}, n=3(*pycard*$^{tpu4/tpu4}$, *pycard*$^{+/+}$); for *pycard* ^{tpu5} , n=6(*pycard*$^{tpu5/tpu5}$, n=6(*pycard*$^{+/+}$). D-E) Based on median gene expression, 20 out of 22 most expressed genes in *pycard*^{tpu4} (n=3) (D) and *pycard*^{tpu5} (n=6) (E) WT fish upon *M. marinum* infection (4 wpi) are found and dominantly expressed in neutrophils according to data by Tang et al. (2017) and accessed via the online tool developed by Lareau et al. 2017. Genes highly expressed in both, *pycard*^{tpu4} and *pycard*^{tpu5} WT fish, are marked with green.

Disease Models & Mechanisms • Supplementary information

Table S1. Differentially expressed genes in the adult *pycard*$^{tpu4/t}$ state in PBS^{pu4} zebrafish at basal injected controls (4 wpi timepoint)

Available for download at
https://journals.biologists.com/dmm/article-lookup/doi/10.1242/dmm.052061#supplementary-data

Table S2. Upregulated genes in the adult *pycard*$^{tpu4/tpu4}$ zebrafish infected with *M. marinum* (4 wpi timepoint)

Available for download at
https://journals.biologists.com/dmm/article-lookup/doi/10.1242/dmm.052061#supplementary-data

Table S3. Downregulated genes in the adult *pycard*$^{tpu4/tpu4}$ zebrafish infected with *M. marinum* (4 wpi timepoint)

Available for download at
https://journals.biologists.com/dmm/article-lookup/doi/10.1242/dmm.052061#supplementary-data

Table S4. Differentially expressed genes in neutrophils of adult *pycard*$^{tpu4/tpu4}$ and *pycard*$^{tpu5/tpu5}$ zebrafish infected with *M. marinum* (4 wpi timepoint)

Available for download at
https://journals.biologists.com/dmm/article-lookup/doi/10.1242/dmm.052061#supplementary-data

Table S5. qPCR primers used in the study

Available for download at
https://journals.biologists.com/dmm/article-lookup/doi/10.1242/dmm.052061#supplementary-data

References

Akl, I., Lelubre, C., Uzureau, P., Piagnerelli, M., Biston, P., Rousseau, A., Badran, B., Fayyad-Kazan, H., Ezedine, M., Vincent, J.-L., et al. (2017). Apolipoprotein L Expression Correlates with Neutrophil Cell Death in Critically Ill Patients. *Shock* **47**, 111–118.

Anes, E., Azevedo-Pereira, J. M. and Pires, D. (2024). Role of Type I Interferons during Mycobacterium tuberculosis and HIV Infections. *Biomolecules* **14**, 848.

Bayés, À., Collins, M. O., Reig-Viader, R., Gou, G., Goulding, D., Izquierdo, A., Choudhary, J. S., Emes, R. D. and Grant, S. G. N. (2017). Evolution of complexity in the zebrafish synapse proteome. *Nat Commun* **8**, 14613.

Bekpen, C., Hunn, J. P., Rohde, C., Parvanova, I., Guethlein, L., Dunn, D. M., Glowalla, E., Leptin, M. and Howard, J. C. (2005). The interferon-inducible p47 (IRG) GTPases in vertebrates: loss of the cell autonomous resistance mechanism in the human lineage. *Genome Biol* **6**, R92.

Boone, D. T., Abdullah, L. and Huang, Y. H. (2023). NDRG3 is a novel regulator of T and B cell development with phosphorylation-specific activity and phenotypes. *The Journal of Immunology* **210**, 219.07-219.07.

Boudinot, P., van der Aa, L. M., Jouneau, L., Du Pasquier, L., Pontarotti, P., Briolat, V., Benmansour, A. and Levraud, J.-P. (2011). Origin and evolution of TRIM proteins: new insights from the complete TRIM repertoire of zebrafish and pufferfish. *PLoS One* **6**, e22022.

Cao, M., Shikama, Y., Kimura, H., Noji, H., Ikeda, K., Ono, T., Ogawa, K., Takeishi, Y. and Kimura, J. (2017). Mechanisms of Impaired Neutrophil Migration by MicroRNAs in Myelodysplastic Syndromes. *The Journal of Immunology* **198**, 1887–1899.

Cardoso, M. S., Gonçalves, R., Oliveira, L., Silvério, D., Téllez, É., Paul, T., Sarrias, M. R., Carmo, A. M. and Saraiva, M. (2024). CD5L is upregulated upon infection with *Mycobacterium tuberculosis* with no effect on disease progression. *Immunology* **173**, 310–320.

Chambers, K. F., Day, P. E., Aboufarrag, H. T. and Kroon, P. A. (2019). Polyphenol Effects on Cholesterol Metabolism via Bile Acid Biosynthesis, CYP7A1: A Review. *Nutrients* **11**, E2588.

Chatzopoulou, A., Heijmans, J. P. M., Burgerhout, E., Oskam, N., Spaink, H. P., Meijer, A. H. and Schaaf, M. J. M. (2016). Glucocorticoid-Induced Attenuation of the Inflammatory Response in Zebrafish. *Endocrinology* **157**, 2772–2784.

Chen, Y., Gao, J., Ma, M., Wang, K., Liu, F., Yang, F., Zou, X., Cheng, Z. and Wu, D. (2024). The potential role of CMC1 as an immunometabolic checkpoint in T cell immunity. *OncoImmunology* **13**, 2344905.

Chestnut, B. and Sumanas, S. (2020). Zebrafish etv2 knock-in line labels vascular endothelial and blood progenitor cells. *Dev Dyn* **249**, 245–261.

Climer, L. K., Cox, A. M., Reynolds, T. J. and Simmons, D. D. (2019). Oncomodulin: The Enigmatic Parvalbumin Protein. *Front Mol Neurosci* **12**, 235.

Denisenko, E., Guler, R., Mhlanga, M., Suzuki, H., Brombacher, F. and Schmeier, S. (2019). Transcriptionally induced enhancers in the macrophage immune response to Mycobacterium tuberculosis infection. *BMC Genomics* **20**, 71.

Du, J., Luo, H., Ye, S., Zhang, H., Zheng, Z. and Liu, K. (2024). Unraveling IFI44L's biofunction in human disease. *Front. Oncol.* **14**, 1436576.

Foulkes, M. J., Henry, K. M., Rougeot, J., Hooper-Greenhill, E., Loynes, C. A., Jeffrey, P., Fleming, A., Savage, C. O., Meijer, A. H., Jones, S., et al. (2017). Expression and regulation of drug transporters in vertebrate neutrophils. *Sci Rep* **7**, 4967.

Funakoshi, S., Shimizu, T., Numata, O., Ato, M., Melchers, F. and Ohnishi, K. (2015). BILL-Cadherin/Cadherin-17 Contributes to the Survival of Memory B Cells. *PLoS ONE* **10**, e0117566.

Georgijevic, S., Subramanian, Y., Rollins, E.-L., Starovic-Subota, O., Tang, A. C. Y. and Childs, S. J. (2007). Spatiotemporal expression of smooth muscle markers in developing zebrafish gut. *Developmental Dynamics* **236**, 1623–1632.

Jiang, H., Tsang, L., Wang, H. and Liu, C. (2021). IFI44L as a Forward Regulator Enhancing Host Antituberculosis Responses. *Journal of Immunology Research* **2021**, 1–12.

Jing, W., Gershan, J. A., Holzhauer, S., Weber, J., Palen, K., McOlash, L., Pulakanti, K., Wesley, E., Rao, S., Johnson, B. D., et al. (2017). T Cells Deficient in Diacylglycerol Kinase ζ Are Resistant to PD-1 Inhibition and Help Create Persistent Host Immunity to Leukemia. *Cancer Res*.

Kajiwara, C., Shiozawa, A., Urabe, N., Yamaguchi, T., Kimura, S., Akasaka, Y., Ishii, Y. and Tateda, K. (2023). Apoptosis Inhibitor of Macrophages Contributes to the Chronicity of *Mycobacterium avium* Infection by Promoting Foamy Macrophage Formation. *The Journal of Immunology* **210**, 431–441.

Kim, G.-D., Das, R., Rao, X., Zhong, J., Deiuliis, J. A., Ramirez-Bergeron, D. L., Rajagopalan, S. and Mahabeleshwar, G. H. (2018). CITED2 Restrains Proinflammatory Macrophage Activation and Response. *Molecular and Cellular Biology* **38**, e00452-17.

Kobayashi, I., Kondo, M., Yamamori, S., Kobayashi-Sun, J., Taniguchi, M., Kanemaru, K., Katakura, F. and Traver, D. (2019). Enrichment of hematopoietic stem/progenitor cells in the zebrafish kidney. *Sci Rep* **9**, 14205.

Kortum, A. N., Rodriguez-Nunez, I., Yang, J., Shim, J., Runft, D., O'Driscoll, M. L., Haire, R. N., Cannon, J. P., Turner, P. M., Litman, R. T., et al. (2014). Differential expression and ligand binding indicate alternative functions for zebrafish polymeric immunoglobulin receptor (pIgR) and a family of pIgR-like (PIGRL) proteins. *Immunogenetics* **66**, 267–279.

Liu, X., Cao, X., Wang, S., Ji, G., Zhang, S. and Li, H. (2017). Identification of Ly2 members as antimicrobial peptides from zebrafish Danio rerio. *Biosci Rep* **37**, BSR20160265.

Lu, X., Nannenga, B. and Donehower, L. A. (2005). PPM1D dephosphorylates Chk1 and p53 and abrogates cell cycle checkpoints. *Genes Dev.* **19**, 1162–1174.

Nishio, H., Suda, T., Sawada, K., Miyamoto, T., Koike, T. and Yamaguchi, Y. (1999). Molecular cloning of cDNA encoding human Rab3D whose expression is upregulated with myeloid differentiation. *Biochim Biophys Acta* **1444**, 283–290.

Ohnishi, K., Melchers, F. and Shimizu, T. (2005). Lymphocyte-expressed BILL-cadherin/cadherin-17 contributes to the development of B cells at two stages. *Eur J Immunol* **35**, 957–963.

Pires, D., Marques, J., Pombo, J. P., Carmo, N., Bettencourt, P., Neyrolles, O., Lugo-Villarino, G. and Anes, E. (2016). Role of Cathepsins in Mycobacterium tuberculosis Survival in Human Macrophages. *Sci Rep* **6**, 32247.

Rao, T., Gao, R., Takada, S., Al Abo, M., Chen, X., Walters, K. J., Pommier, Y. and Aihara, H. (2016). Novel TDP2-ubiquitin interactions and their importance for the repair of topoisomerase II-mediated DNA damage. *Nucleic Acids Res* gkw719.

Rong, C., Shi, Y., Huang, J., Wang, X., Shimizu, R., Mori, Y., Murai, A. and Liang, J. (2020). The Effect of Metadherin on NF-κB Activation and Downstream Genes in Ovarian Cancer. *Cell Transplant* **29**, 963689720905506.

Sanjurjo, L., Aran, G., Téllez, É., Amézaga, N., Armengol, C., López, D., Prats, C. and Sarrias, M.-R. (2018). CD5L Promotes M2 Macrophage Polarization through Autophagy-Mediated Upregulation of ID3. *Front. Immunol.* **9**, 480.

Schindelin, J., Arganda-Carreras, I., Frise, E., Kaynig, V., Longair, M., Pietzsch, T., Preibisch, S., Rueden, C., Saalfeld, S., Schmid, B., et al. (2012). Fiji: an open-source platform for biological-image analysis. *Nat Methods* **9**, 676–682.

Somoza, R. and Beutler, E. (1983). Phosphoglycolate phosphatase and 2,3-diphosphoglycerate in red cells of normal and anemic subjects. *Blood* **62**, 750–753.

Tokuhisa, M., Kadowaki, T., Ogawa, K., Yamaguchi, Y., Kido, M. A., Gao, W., Umeda, M. and Tsukuba, T. (2020). Expression and localisation of Rab44 in immune-related cells change during cell differentiation and stimulation. *Sci Rep* **10**, 10728.

Varela, M., Diaz-Rosales, P., Pereiro, P., Forn-Cuní, G., Costa, M. M., Dios, S., Romero, A., Figueras, A. and Novoa, B. (2014). Interferon-Induced Genes of the Expanded IFIT Family Show Conserved Antiviral Activities in Non-Mammalian Species. *PLoS ONE* **9**, e100015.

Veneman, W. J., Stockhammer, O. W., de Boer, L., Zaat, S. A. J., Meijer, A. H. and Spaink, H. P. (2013). A zebrafish high throughput screening system used for Staphylococcus epidermidis infection marker discovery. *BMC Genomics* **14**, 255.

Xin, G.-Y., Li, W.-G., Suman, T. Y., Jia, P.-P., Ma, Y.-B. and Pei, D.-S. (2020). Gut bacteria Vibrio sp. And Aeromonas sp. trigger the expression levels of proinflammatory cytokine: First evidence from the germ-free zebrafish. *Fish & Shellfish Immunology* **106**, 518–525.

Xu, S., Xie, F., Tian, L., Manno, S. H., Manno, F. A. M. and Cheng, S. H. (2019). Prolonged neutrophil retention in the wound impairs zebrafish heart regeneration after cryoinjury. *Fish Shellfish Immunol* **94**, 447–454.

Zhou, Z. and Sun, L. (2016). Edwardsiella tarda-Induced Inhibition of Apoptosis: A Strategy for Intracellular Survival. *Frontiers in Cellular and Infection Microbiology* **6**, 76.
